# Antagonistic action of GPS2 and KDM1A at enhancers governs alternative macrophage activation by interleukin 4

**DOI:** 10.1093/nar/gkac1230

**Published:** 2023-01-05

**Authors:** Zhiqiang Huang, Astradeni Efthymiadou, Ning Liang, Rongrong Fan, Eckardt Treuter

**Affiliations:** Department of Biosciences and Nutrition, Karolinska Institutet, 14183 Huddinge, Sweden; Department of Biosciences and Nutrition, Karolinska Institutet, 14183 Huddinge, Sweden; Department of Biosciences and Nutrition, Karolinska Institutet, 14183 Huddinge, Sweden; Department of Biosciences and Nutrition, Karolinska Institutet, 14183 Huddinge, Sweden; Department of Biosciences and Nutrition, Karolinska Institutet, 14183 Huddinge, Sweden

## Abstract

The Th2 cytokine interleukin 4 (IL4) promotes macrophage differentiation into alternative subtypes and plays important roles in physiology, in metabolic and inflammatory diseases, in cancer and in tissue regeneration. While the regulatory transcription factor networks governing IL4 signaling are already well-characterized, it is currently less understood which transcriptional coregulators are involved and how they operate mechanistically. In this study, we discover that G protein pathway suppressor 2 (GPS2), a core subunit of the HDAC3 corepressor complex assembled by SMRT and NCOR, represses IL4-dependent enhancer activation in mouse macrophages. Our genome-wide and gene-specific characterization revealed that, instead of directly repressing STAT6, chromatin-bound GPS2 cooperates with SMRT and NCOR to antagonize enhancer activation by lysine demethylase 1A (KDM1A, LSD1). Mechanistically, corepressor depletion increased KDM1A recruitment to enhancers linked to IL4-induced genes, accompanied by demethylation of the repressive histone marks H3K9me2/3 without affecting H3K4me1/2, the classic KDM1A substrates for demethylation in other cellular contexts. This in turn caused enhancer and gene activation already in the absence of IL4/STAT6 and sensitized the STAT6-dependent IL4 responsiveness of macrophages. Thus, our work identified with the antagonistic action of a GPS2-containing corepressor complex and the lysine demethylase KDM1A a hitherto unknown epigenetic corepressor-coactivator switching mechanism that governs alternative macrophage activation.

## Graphical Abstract

**Graphical Abstract:**
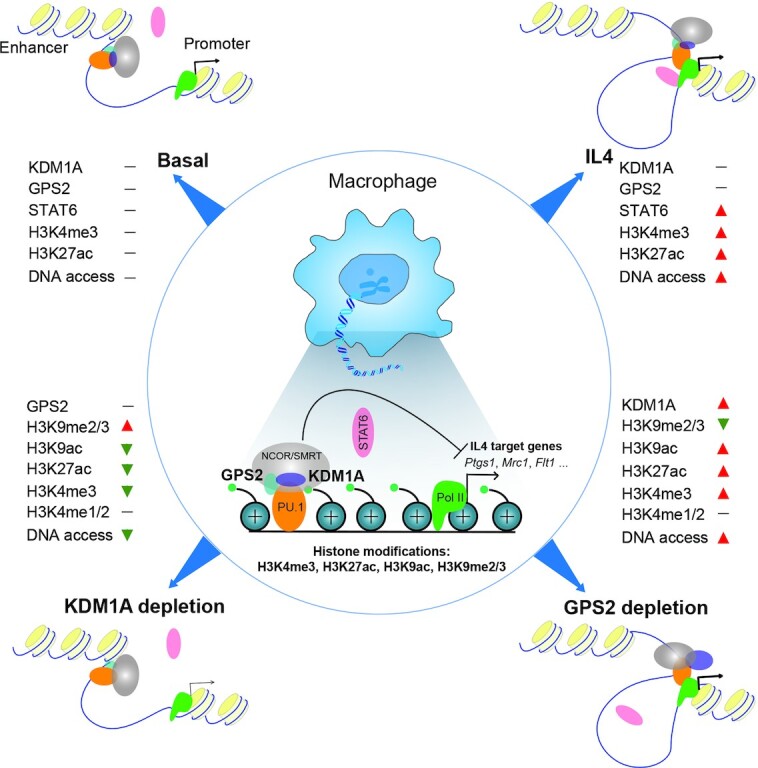
A GPS2-containing corepressor complex and the lysine demethylase KDM1A (LSD1) crosstalk during interleukin 4-mediated alternative macrophage activation to antagonistically regulate histone modifications, enhancer activity and gene expression.

## INTRODUCTION

Macrophages are crucial components of innate immunity that are present in almost all tissues. Their functionality and activation states are tightly regulated by tissue-derived signals. Based on their inflammatory properties, macrophages are classified into distinct activation states, from pro-inflammatory M1 to anti-inflammatory M2 subtypes (1–4). Th2-type cytokines such as interleukin 4 (IL4) promote the differentiation of M2 macrophages and thereby control multiple physiological processes ranging from immune modulation in obesity, cardiovascular diseases and type 2 diabetes to wound healing and tissue regeneration (5–8). IL4-remodeled M2 macrophages are generally considered beneficial as they alleviate chronic metabolic inflammation and improve energy balance in tissues. In contrast, in tumor-associated macrophages, IL4 creates an inflammation inhibitory microenvironment which in turn drives tumor growth and metastasis (9,10). Therefore, deciphering the multifaceted roles of IL4 signaling in macrophages is also crucial for better understanding mechanisms underlying human diseases.

Previous studies, using bone marrow-derived macrophages (BMDMs) and the macrophage cell line RAW264.7 as common experimental models, have identified several transcription factors (TFs) that are critical in regulating IL4 pathways in mice (11–16). Studies using monocyte-to-macrophage differentiation have revealed that the requirement of the same TF classes appears conserved in human IL4 pathways (4,17). These studies suggest that the primary IL4-regulated TF is STAT6, which induces early IL4 responses along with inducing PPARγ and EGR2 to control late IL4 responses (11–13,18). These signal-responsive TFs (SRTFs) cooperate with lineage-determining TFs (LDTFs) such as PU.1/ETS factors, AP1 family members (JUN, JUNB, FOS), CEBPs and MYC to regulate chromatin accessibility, enhancer activity and IL4 target gene expression. This cooperation is highly dynamic and influenced by cellular context, determining TF expression and signal responses, and individual genetic variation of DNA sequence motifs which dictate the TF association with chromatin. These aspects likely contribute to the variation of IL4 responses in individuals, as demonstrated for example by the substantial differences of IL4-regulated gene expression in five mouse strains carrying natural genetic variants, some of which affecting also STAT6 binding (11). Moreover, stimulation of BMDMs with IL4 and interferon γ (IFNγ) revealed extensive transcriptional and epigenomic crosstalk between M1 and M2 pathways, emphasizing that the participating SRTFs and LDTFs influence each other to control gene expression under complex environmental conditions (16).

Beyond TFs and genetic variation controlling IL4 signaling, above studies also indicated that there is another level of regulation related to the control of gene expression by epigenetic mechanisms. Transcriptional coregulators are suspected to play key roles in these mechanisms, as they are required for TFs to modulate the chromatin landscape and to control gene transcription (19,20). Alterations in the ratio of TFs and associated corepressors and coactivators, often acting in complexes that carry histone-modifying activities such as deacetylases and demethylases, cause epigenetic changes within specific chromatin regions defined by topologically associating domains (TADs). Within individual TADs, TF-coregulator-networks operate to regulate mRNA transcription rates (gene expression) by binding to cis-regulatory elements (CREs), including enhancers, silencers and promoters, by stimulating or inhibiting eRNA transcription and enhancer-promoter looping, by modifying specific histone marks linked to activation or repression, and by communicating with the basal RNA polymerase II machinery (21–26).

In contrast to the evidence already provided for TFs and enhancers, the epigenetic regulatory mechanisms by which coregulators, especially corepressors, participate in macrophage M2 activation are currently poorly characterized. Among the relevant candidates is a fundamental corepressor complex containing the core subunits histone deacetylase 3 (HDAC3), G protein pathway suppressor 2 (GPS2), nuclear receptor corepressor 1 (NCOR, alias N-CoR, NCOR1), silencing mediator of retinoid and thyroid hormone receptors (SMRT, alias NCOR2), and two transducing beta-like proteins (TBL1, TBLR1) (20,27–31). Notably, macrophage-specific knockout (KO) mice depleting different subunits of the complex show in part opposite inflammatory and metabolic phenotypes, suggesting the existence of sub-complexes or modules (20). NCOR or HDAC3 seem to partly control IL4-induced gene expression (32–34), and also to participate in IL4-dependent repression of gene subsets (15). SMRT (encoded by the human gene *NCOR2*) has additionally been shown to be critical for IL4-dependent human monocyte differentiation (17). Our studies have particularly revealed that SMRT and GPS2 depletion sensitizes mouse macrophages to pro-inflammatory stimuli, consistent with correlation analysis of human adipose tissue macrophages in the context of obesity and type 2 diabetes (35,36). This was mechanistically further explored with an emphasis on enhancers and silencers that control pro-inflammatory gene expression in BMDMs and RAW264.7 cells (37). Although the corepressor complex seems well positioned to integrate both M1 and M2 signals, it remains to be elucidated how it mechanistically controls the macrophage IL4 response in a gene-selective manner, in cooperation with TFs and potentially other coregulators.

In this study, we systemically investigate these issues with specific focus on the corepressor complex subunit GPS2, along with studying the involvement of SMRT and NCOR. Our data indicate that GPS2, as integral part of the corepressor complex, represses IL4 target gene transcription by modulating enhancer-promoter looping, chromatin accessibility and histone modifications, but without directly interacting with STAT6. In search for other alternative mechanisms, we identified the histone demethylase KDM1A (also known as LSD1) using chromatin proteomics. As so far neither GPS2 nor KDM1A have been linked to the M2 pathway, we dissected their mechanistic relationship both genome-widely and at individual IL4 target genes. We show that GPS2 depletion increased KDM1A recruitment to enhancers linked to IL4-induced genes, accompanied by demethylation of the repressive histone marks H3K9me2/3 without affecting H3K4me1/2, the classic KDM1A substrates. This in turn caused enhancer and gene activation already in the absence of IL4/STAT6 and sensitized the STAT6-dependent IL4 responsiveness of macrophages. We therefore discovered a hitherto unknown epigenetic mechanism by which the antagonistic action of the corepressor GPS2 and the coactivator KDM1A controls IL4-dependent macrophage activation and maintains a repressive basal state prior to IL4 activation. Because GPS2 expression and function varies in human metabolic-inflammatory disease states, and KDM1A can be pharmacologically modulated, the identified antagonism could be of both mechanistic and translational importance.

## MATERIALS AND METHODS

### Macrophage cell cultures and treatments

The mouse macrophage cell line RAW264.7, hereafter referred to as RAW cells, was purchased from ATCC (ATCC, TIB-71). Cells were cultured in DMEM medium supplemented with 10% FBS and 100 U/ml pen/strep. The *Gps2* KO RAW cell line was generated using CRISPR-Cas9 as described previously (36,37). For RNA-seq and RT-qPCR analysis, RAW cells were incubated with IL4 (20 ng/ml) for 6 h before RNA extraction. PPAR$\gamma$ ligand (Rosiglitazone, 5 μM) treatment was for 12 h. For ChIP-seq, CUT&Tag, ATAC-seq and 4C-seq experiments, RAW cells were subjected to 1 h IL4 (20 ng/ml). For PU.1 inhibition experiments, 5 μM of DB2115 (MCE, HY124676A) inhibitor (38) was used overnight to block the DNA binding of PU.1 in RAW cells. For KDM1A (LSD1) inhibition experiments, RAW cells were incubated overnight with 1 μM of the indicated inhibitors GSK-LSD1 (Sigma, SML1072), LSD1i-S2101 (Sigma, 489477) and LSD1i-SP2509 (Sigma, 5.09703). Protein samples were extracted for western blot (H3K4me1/2). GSK-LSD1 was used to check the enrichment of H3K4me1/2 and KDM1A at the *Ptgs1* locus by ChIP-qPCR. GSK-LSD1 was also used to measure the gene expression in GPS2 KO RAW cells. Bone Marrow-Derived Macrophages (BMDMs) were isolated from femur or tibia of macrophage-specific *Gps2* KO mice (C57Bl/6 background) as described in our previous studies (36,37). BMDMs were differentiated in DMEM medium supplemented with 10% FBS, 100 U/ml pen/strep and 30% L929 conditioned medium for 7 days before further experiments. Differentiated BMDMs were treated with IL4 (20 ng/ml) for 6 h for RT-qPCR or RNA-seq analysis. Human blood CD14^+^ monocytes were obtained from Lonza (Catalog: 4W-400). Monocytes were maintained in X-VIVO medium (Lonza, BE04-448Q) supplemented with 10% FBS and 20 ng/ml GM-CSF (Sigma, G5035) for one week differentiation into macrophages. Differentiated macrophages were subjected to *GPS2* knockdown and control lentivirus infection and selected with 1 μg/ml puromycin for two additional days. GPS2-depleted and control macrophages were treated with human IL4 (20 ng/ml) (Sigma, GF337) for 6 h, and gene expression was determined by RT-qPCR. HEK293 (ATCC, CRL-1573) cells were maintained in DMEM medium supplemented with 10% FBS and pen/strep 100 U/ml and used for lentivirus packaging and co-immunoprecipitation experiments.

### Lentivirus-shRNA-mediated knockdown in RAW cells

Lentiviral shRNA sequences were designed via GPP Web Portal (Broad Institute). Mouse targets: *Gps2* (clone ID: TRCN0000037133), *Ncor (Ncor1)* (clone ID: TRCN0000350169), *Smrt (Ncor2)* (clone ID: TRCN0000238140), *Stat6* (clone ID: TRCN0000226179), *Pparg* (clone ID: TRCN0000001658), and *Kdm1a* (Clone ID: TRCN0000071375). Human target: *GPS2* (clone ID: TRCN0000036876). The shRNA sequences were synthesized and constructed into the PLKO.1-TRC vector (Addgene, 10878). The lentiviral shRNAs were then packaged in HEK293FT cells using psPAX2 (Addgene, 12260) and pMD2.G (Addgene, 12259). The lentiviral particles were transduced into RAW macrophages and stable cell lines were generated using 5 μg/ml puromycin selection for 5 days. The stable cell lines were expanded and used for further experiments.

### Western blot analysis

2 × 10^5^ cells were seeded one day before the experiments and washed twice with PBS before they were lysed in RIPA buffer. Protein concentrations were determined by BCA assay. Protein samples were resolved for SDS-PAGE, blotted onto PVDF membranes and probed with antibodies for PTGS1 (Sigma, AV41836), GPS2 (custom-made rabbit polyclonal IgG, as previously described (36,39), NCOR (Bethyl, A301-145A), SMRT (Bethyl, A301-147A), H3 (Abcam, ab1791), STAT6 (Sigma, S-6433), p-STAT6 (Thermo, 700247), β-actin (Abcam, ab8226), H3K27ac (Abcam, ab4729), H3K4me3 (Abcam, ab8580), H3K9me2 (Abcam, ab1220), H3K9me3 (Abcam, ab8898) or KDM1A (Abcam, ab17721). Membranes were developed using the ECL system. All antibodies are listed in the Supplementary Table II.

### Co-immunoprecipitations

HEK293 (ATCC, CRL-1573) cells were co-transfected with pcDNA3-HA-GPS2 (36) or pCMV3-HA-KDM1A (40) and expression plasmids for the indicated FLAG-tagged TFs (36). Cells were lysed after 48 h transfection, and the lysate was incubated with anti-HA (BioLegend, 901513) or anti-FLAG (Sigma-Aldrich, F7425) coupled to protein A magnetic beads for 3 h at 4°C. Beads were washed with lysis buffer five times and eluted at 98°C for 10 min. The eluted sample was used for western blot and detected with anti-FLAG or anti-HA antibodies. Whole-cell lysate was used as input.

### ATAC-seq

ATAC-seq (Assay for Transposase-Accessible Chromatin using sequencing) using cell nuclei was performed as described previously (37,41). RAW cells were treated with vehicle or IL4 (20 ng/ml) for 1 h and washed with PBS twice. The cells were then scrapped and resuspended in lysis buffer. Cell nuclei were spun down and transferred into transposition reaction buffer and incubated at 37°C for 30 min. Genomic DNA was extracted using PCR Purification Kit (Qiagen, 28106). ATAC-seq library amplification was performed with the previously reported protocol (41). The purified DNA library mix was sequenced on NextSeq 550 (Illumina, 75 SE reads) in BEA Core Facility (Karolinska Institutet, Huddinge, Sweden) with pair-ended output.

### Rapid immunoprecipitation mass spectrometry of endogenous proteins (RIME)

The RIME in BMDMs and RAW cells was performed according to a previous protocol with slight modifications (42). In brief, four plates (15 cm) of RAW/BMDM cells were first crosslinked with 10 ml 2 mM disuccinimidyl glutarate (DSG) for 30 min and then with 10 ml 1% formaldehyde for 10 min. The crosslinking was terminated by adding 500 μl 2.5 M of glycine to the cells and incubation for 5 min. The cells were then washed three times with cold PBS containing protease inhibitors. Cells were lysed in 50 ml lysis buffer with 50 mM HEPES–KOH (pH 7.5), 140 mM NaCl, 1mM EDTA, 10% glycerol, 0.5% Igepal CA-630, 0.25% Triton X-100 for 10 min, the nuclei of the cells were then pelleted with centrifugation at 4200 rpm at 4°C for 10 min. The nuclei were resuspended in 40 ml buffer with 10 mM Tris–HCl (pH 8.0), 200 mM NaCl, 1 mM EDTA, 0.5 mM EGTA for 10 min and pelleted at 4200 rpm at 4°C for 10 min. The nuclei pellets were resuspended with 8 ml buffer containing 10 mM Tris–HCl (pH 8.0), 100 mM NaCl, 1mM EDTA, 0.5mM EGTA, 0.1% Na-deoxycholate, 0.5% *N*-lauroylsarcosine. The lysed nuclear extracts were sonicated for 35 cycles (30 s ON/30 s OFF) in Bioruptor Pico. For immunoprecipitation, 2 μg of rabbit polyclonal GPS2 antibody (custom-made, as described previously (36,43), or IgG were incubated with 25 μl Protein A Dynabeads (Invitrogen, 10002D) in 1 ml blocking buffer (0.5% BSA) overnight. The beads were then washed three times with cold blocking buffer and incubated with 100 μg chromatin at 4°C overnight with slow rotation. After the incubation, the beads were washed 6–7 times with wash buffer containing 50 mM HEPES–KOH (pH 7.5), 500 mM LiCl, 1 mM EDTA, 1% NP-40, 0.7% Na-deoxycholate. To enrich the immunoprecipitated proteins, we merged six samples and used 20 μl PAGE gel loading buffer to elute proteins. The samples were boiled at 95 $^\circ$C for 10 min. The beads were removed using a magnet stand. The eluted samples were separated by protein electrophoresis for about 5 min using 100 V to remove the detergents. The gel pieces containing the eluted protein samples were cut out and transferred into 1.5 ml tubes and stored in 1 ml distilled water. Protein samples were subjected to LC–MS/MS (Proteomics core facility, ZMBH Heidelberg, Germany). The identified interaction protein matrix was filtered by comparing the enrichment with the IgG groups. The matrix was further uploaded to the online SRING platform (44) to search the interaction networks.

### ChIP-seq, ChIP-qPCR and CUT&Tag

Chromatin immunoprecipitation followed by sequencing (ChIP-seq) and qPCR (ChIP-qPCR) were performed as previously described (35–37). The following antibodies were used: H3K27ac (Abcam, ab4729), H3K4me1 (Abcam, ab176877), H3K4me2 (Abcam, ab32356), H3K4me3 (Abcam, ab8580), GPS2 (custom-made, as described previously (36,43), Pol II (BioLegend, 8WG16), NCOR (Bethyl, A301-145A), SMRT (Bethyl, A301-147A), STAT6 (Cell Signaling, 9362), pSTAT6 (Thermo, 700247), KDM1A (Abcam, ab17721), H3K9me2 (Abcam, ab1220), H3K9me3 (Abcam, ab8898) and H3K9ac (Sigma, 06–942). All ChIP-seq antibodies are listed in the Supplementary Table II. Briefly, one 15 cm^2^ plate of RAW cells were crosslinked with 10 ml 2 mM disuccinimidyl glutarate (DSG) (VWR, A7822.0001) for 30 min followed by 10 ml 1% formaldehyde for 10 min. The crosslink reaction was stopped by adding 500 μl 2.5 M glycine to the final concentration of 0.125 M and incubating for 5 min. The lysed RAW cell nuclei were sonicated for 30 min (30s ON/30s OFF) with Bioruptor Pico (Diagenode, B01060010). Protein A Dynabeads (Invitrogen, 10002D) were incubated with indicated antibodies (1–4 μg). The ChIP-ed DNA was purified using Clean & Concentrator Capped Zymo-Spin I (Zymo Research, D4013) kit. The DNA was used for ChIP-qPCR using primers against the indicated regions (Supplementary Table I) and for the library preparation. The ChIP-seq library was prepared using Takara ThruPLEX DNA-Seq Kit (Takara, R400736). Sequencing was performed in the Illumina NextSeq 550 (Illumina, 75SE reads) by the BEA Core Facility (Karolinska Institutet, Huddinge, Sweden) with single-ended output. ChIP-qPCR was performed with SYBR Green (KAPA Biosystems, 07959567001) to validate the ChIP-seq results. CUT&Tag was performed using a published protocol (45). Library samples were sequenced on Novaseq 6000 (S4, PE150) and NextSeq 2000 (PE100) platforms using pair-ended output.

### Computational analysis of ChIP-seq, CUT&Tag and ATAC-seq data

The public ChIP-seq data GSM4848501, GSM4848502, GSM4848505, GSM4848506, GSM2845662, GSM2845663, GSM2845664, GSM2845665, GSM2867738, GSM2867739, GSM1631862 and GSM1631866 were obtained from GEO platform and re-analyzed by standard protocols. All public ChIP-seq datasets are listed in the Supplementary Table III. Sequencing files in this study (FASTQ files) were aligned to the NCBI37/mm9 version of the mouse reference genome, using Bowtie on the Uppsala Multidisciplinary Center for Advanced Computational Science (UPPMAX) under project SNIC2018/8-122. The sequencing tags were then read and imported to HOMER (46). Peaks were identified using HOMER with default settings (the peak calling parameters were slightly different between ChIP-seq for histone marks and TFs/coregulators, and overlapped peaks were calculated by merging together all individual peak files from each experiment).The CUT&Tag data peak calling was performed using MACS2 (47). The statistical comparison of differential peak tag counts was performed using edgeR in R. Peak changes with an adjusted *P*-value <0.05 were considered as differential peaks. Bedtools was used to find the overlapped peaks for the indicated analysis and extracted the desired peaks coordinates (GPS2 etc.) Peak coverage analysis was perform using annotatePeaks.pl and plot the 3 kb upstream and 3 kb downstream region from the indicated peak center. Heatmap plot was performed using Deeptools (48). The analysis of ATAC-seq results was described in our previous study (37). Paired-end data were aligned to the mouse mm9 genome using Bowtie2 and ATAC-seq peak calling was done using MACS2 (47). The statistical analysis for differential expression was further performed using edgeR. Peak changes with an adjusted *P*-value <0.05 were considered as differential peaks. In the quantitative analysis (percentage) for both ChIP-seq and CUT&Tag, total tags (10^7^) were used as the normalization factor. The related datasets were used from the HOMER outputs.

### RNA isolation, RT-qPCR and RNA-seq

Total RNA was extracted from the cells using the E.Z.N.A. Total RNA Kit (Omega) according to the manufacturer instructions. One microgram of total RNA was used for reverse transcription using Superscript II reverse transcriptase kit (Life Technologies, 18064022). *Gapdh/GAPDH/RPS14* was used as internal control. Relative changes in mRNA expression were calculated using the comparative cycle method (2^−ΔΔCt^). The RT-qPCR primers are listed in the Supplementary Table I. RNA-seq samples for both *Gps2* KO and KDM1A knockdown cells were sent to Novogene and the BEA Core Facility (Karolinska Institutet, Sweden) for library preparation and sequencing. RNA-seq data from WT vs. *Stat6* KO BMDMs and the related STAT6 ChIP-seq data were obtained from the public GEO dataset GSE106706 (15). The FASTQ data were aligned to mouse mm9 genome using HISAT2 and Bowtie2 on Galaxy platform. The re-analysis of the RNA-seq was performed by HOMER software (46). Transcripts with an adjusted *P*-value <0.05 were considered as differentially expressed genes. Gene tag counts were extracted by -rpkm normalization and visualized in GraphPad software and with ggplot2 (version 3.3.6). The Gene Ontology (GO) enrichment analysis was performed with the R package clusterProfiler (version 4.4.4) (49) and the enriched terms were visualized as a network with the same package. The R package DoRothEA (version 1.8.0) was used as a source of TF-gene target interaction information (50). Only the interactions with confidence level A, B and C were kept. The viper algorithm of this package was used to perform the enrichment analysis of the target's expression in the RNA-seq data. The minimum number of targets per TF was set to 5 and the eset.filter to FALSE.

### 4C-seq and data analysis

The circular chromosome conformation capture assay followed by high-throughput sequencing (4C-seq) was modified from a previous report (51) and executed according to the detailed protocol described in our previous study (52). In brief, two 15 cm^2^ RAW cells were crosslinked with 2% formaldehyde for 10 min and stopped with 0.125 M glycine (final concentration). Cells were counted and divided into 10^7^ cells per tube. The cells were re-suspended in 10 ml cytosol lysis buffer containing 50 mM Tris–HCl, 150 mM NaCl, 5 mM EDTA, 0.5% NP-40, 1% Triton X-100 for 10 min. The nuclei were pelleted and resuspended with 440 μl Milli-Q water. 60 μl digest buffer, 15 μl 10% SDS and 75 μl 20% Triton X-100 were added to the cell nuclei and the mix was incubated at 37°C for 2 h, with 900 rpm shaking speed. After that, 200 U of the restriction enzyme DpnII (NEB, R0543M) was added for three times and the sample mix was kept in the shaking incubator overnight. The enzyme was inactivated by incubating for 20 min at 65°C. Samples were transferred to 50 ml Falcon tubes with 700 μl ligation buffer and 5.5 ml Milli-Q water. 100 U T4 ligase (NEB, M0202M) was added and the mix was incubated overnight at room temperature. The ligated genomic DNA was purified by phenol-chloroform buffer and further digested with the second enzyme BfaI (NEB, R0568L) at 37°C overnight. The second restriction enzyme was inactivated by incubating the mix for 20 min at 65°C and the secondary ligation was done at room temperature overnight. The secondary ligated DNA was purified by QIAquick PCR purification kit (QIAGEN, 28104). The 4C library was amplified by *Ptgs1* 4C promoter bait primers using expand long template PCR system (Roche, 117590600001). The 4C-PCR primer was listed in the Supplementary Table I. The amplified DNA was purified by QIAquick PCR purification kit. DNA concentrations were determined by Qubit Fluorometric Quantification kit (ThermoFisher, Q33238). 10 ng of DNA was used for the high-throughput sequencing library preparation. The library was prepared using SMARTer PicoPLEX library preparation kits (Takara, R400676) and SMARTer DNA unique dual index kits (Takara, R400661). The purified DNA library mix was sequenced using NextSeq 550 (Illumina,75SE reads) at the BEA Core Facility (Karolinska Institutet, Sweden). The FASTQ data were generated by the standard 4C analysis of 4Cseqpipe protocol (51). The reading primer sequences were trimmed from the raw reads. The *cis*-interacting DNAs were plotted in the same chromosome window. All data were normalized to the same read counts for comparative analysis.

### Statistical analysis

All experiments were done with biological replicates and were performed at least two times. Statistical tests were performed using GraphPad Prism 8.0 (GraphPad Software, Inc., La Jolla, CA), and all data are represented as mean ± s.e.m. Normal distribution tests were performed before statistical analysis. Group comparisons were assessed by Student's *t*-test (two groups), and one-way ANOVA test followed by Tukey's or Dunnett's post hoc test (multiple comparisons). Hypergeometric test (phyper) was used to check the data correlation between *Gps2* KO and KDM1A knockdown. All statistical tests were two-tailed, and *P* <0.05 was defined as significant. No statistical methods were used to predetermine sample size. No samples were excluded from the analyses.

## RESULTS

### GPS2 depletion sensitizes macrophages to IL4 treatment by enhancer de-repression

**Figure 1.**
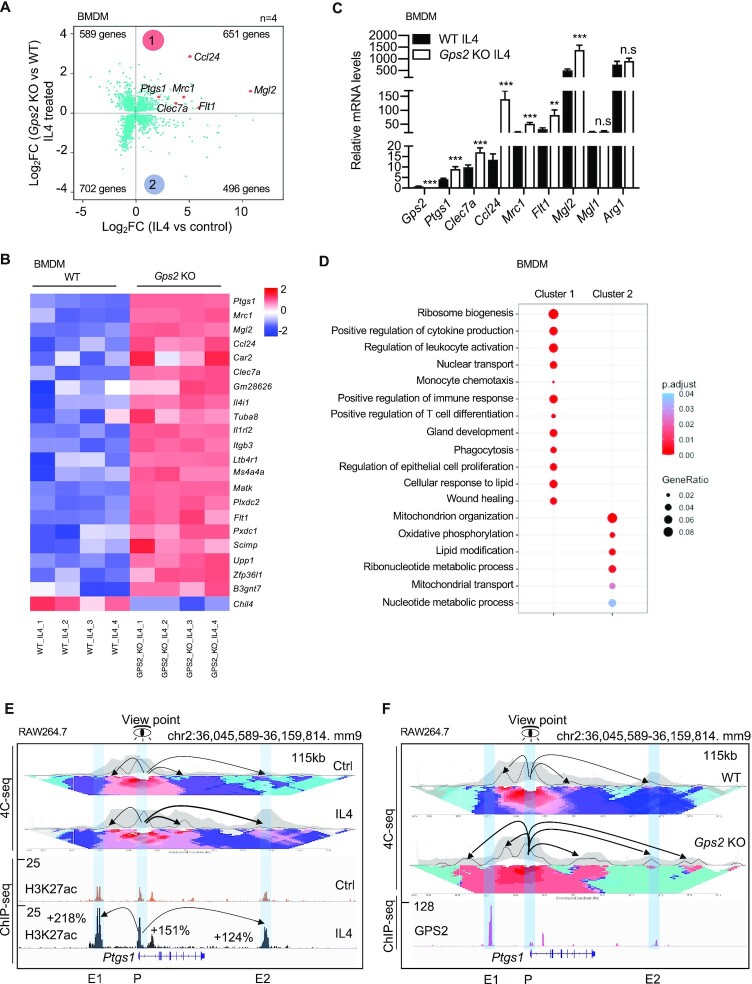
(**A**) Scatter plot showing the comparison of transcriptome signatures between *Gps2* KO and IL4 treatment in BMDMs. The X-axis represents the gene log_2_-fold changes in IL4 versus control, and the Y-axis represents the gene log_2_-fold changes in IL4-treated *Gps2* KO versus WT BMDMs. All significantly changed genes are presented in each quadrant. Several IL4-inducible GPS2 target genes are highlighted in red. (**B**) Heatmap representing the top 20 up-regulated (based on the adj *P*-value) genes in IL4-treated *Gps2* KO versus WT BMDMs. (**C**) RT-qPCR analysis of mRNA expression in IL4-treated WT and *Gps2* KO BMDMs (*n* = 4). Unpaired *t*-test was used for the group comparison. (**D**) Go over-representation analysis of selected representative processes for cluster 1 and cluster 2 in *Gps*2 KO BMDMs. (**E**) Representative image showing the tracks of 4C-seq and ChIP-seq-based contact profiles at the *Ptgs1* locus with IL4 treatment in RAW cells using the *Ptgs1* promoter region as bait (Viewpoint). The upper panel shows the interaction regions and profiles set to a window using 20–80% percentile values. The interaction frequencies were normalized to the strongest point (bait region) and presented based on a color-coded scale. The lower panel shows the H3K27ac changes upon IL4 treatment (1 h). The percentage changes are highlighted. (**F**) Representative image showing the track of 4C-seq contacts at the *Ptgs1* locus in *Gps2* KO RAW cells using the *Ptgs1* promoter (P) region as bait (basal condition). GPS2 ChIP-seq in the lower panel is used to mark the enhancer (E1, E2) and promoter (P) regions. Unpaired *t-*test was used to determine data significance. All data are represented as mean ± s.e.m. *P < 0.05, **P < 0.01, ***P < 0.001.

To explore the involvement of GPS2 in macrophage IL4 pathways, we performed RNA-seq in bone marrow-derived macrophages (BMDMs) of WT and *Gps2* KO mice with or without IL4 stimulation (Figure 1A). Depletion was specific to GPS2 and had no effect on other core subunits of the corepressor complex (Supplementary Figure S1A). We then compared the transcriptome signatures of IL4 treatment and *Gps2* KO in BMDMs (Figure 1A). Around 60% of IL4-activated genes were de-repressed by GPS2 depletion (labelled as cluster 1 in Figure 1A) in contrast to the other 40% genes (cluster 2) that were decreased in the *Gps2* KO macrophages. Among the GPS2-repressed IL4 target genes were classical M2 macrophage markers such as *Ptgs1, Mrc1, Mgl2, Clec7a* (Figure 1B). The RNA-seq results were validated using RT-qPCR (Figure 1C). The regulation by GPS2 was gene-selective because IL4-induced *Arg1* expression was not changed in *Gps2* KO BMDMs (Figure 1C). The gene ontology analysis in cluster 1 and 2 showed that GPS2-repressed IL4 target genes were functionally linked to inflammatory and defense responses while GPS2-activated IL4 target genes mainly controlled mitochondrial and energy metabolism (Figure 1D and Supplementary Figure S1B). The transcriptome signature regulated by GPS2 and IL4 was conserved between BMDMs and the macrophage cell line RAW264.7 (hereafter referred as RAW cells) (Supplementary Figure S1C and S1D). GPS2 repression of IL4 signature genes appears to be conserved also in human CD14^+^ monocyte-derived macrophages, because GPS2 depletion increased IL4-induced expression of *PTGS1*, *CLEC7A*, *CCL24*, *MRC1*, *FLT1* and *CLEC10A* (relates to mouse *Mgl2*) (Supplementary Figure S1E).

RAW cells were used to further investigate the molecular mechanisms. Using ChIP-seq of H3K27ac (marking active enhancers and promoters, epigenome) and GPS2 (marking corepressor/TF binding sites, cistrome) in RAW cells we mapped putative enhancers and promoters at all IL4 target gene loci, exemplified for *Ptgs1* consisting of one upstream (E1) and one downstream (E2) enhancer (Figure 1E, F). 4C-seq revealed that interaction of both enhancers with the *Ptgs1* promoter was increased by either IL4 treatment (Figure 1E) or GPS2 depletion (Figure 1F) in a similar way. This indicates that reduced GPS2 expression can trigger alternative routes of IL4 target gene activation in the absence of IL4/STAT6 induction. Overall, we conclude that enhancer-bound GPS2 directly represses IL4 target genes via preventing enhancer-promoter looping and H3K27 acetylation.

### GPS2 cooperates with NCOR and SMRT to regulate IL4 target gene expression

**Figure 2.**
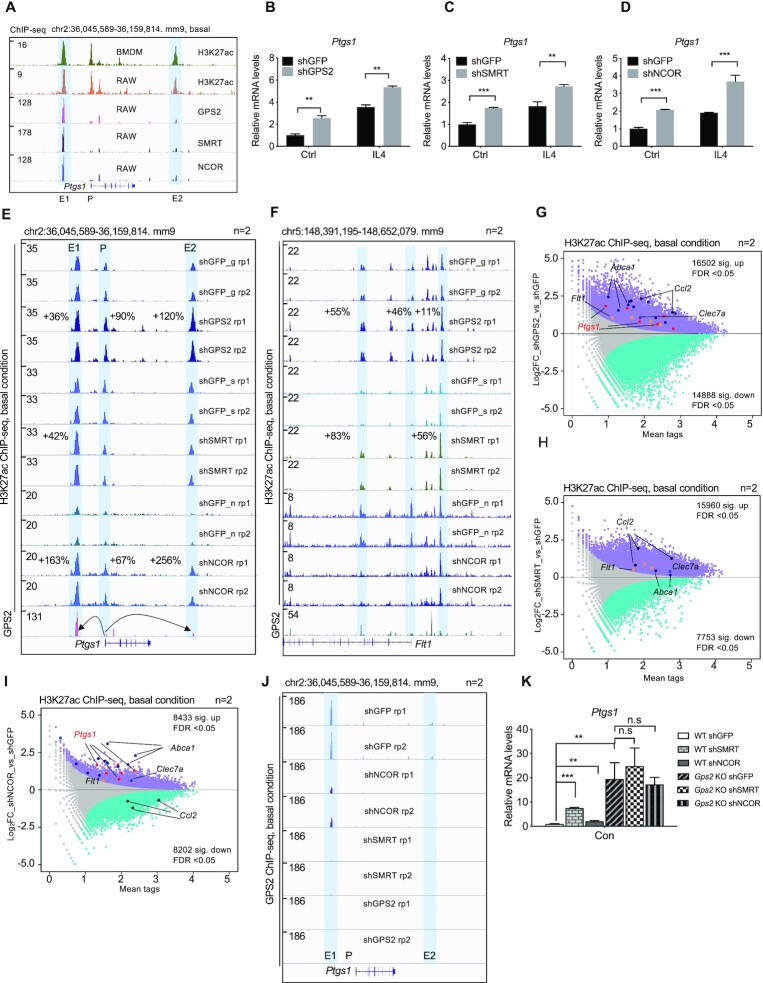
(**A**) IGV genome browser tracks representing the GPS2, NCOR, SMRT and H3K27ac ChIP-seq peaks at the *Ptgs1* locus in both BMDMs and RAW cells. Enhancer (E1, E2) and promoter (P) regions are highlighted. (**B–D**) RT-qPCR showing the mRNA expression of *Ptgs1* in lentivirus shRNA-mediated GPS2, SMRT and NCOR knockdown RAW cells (*n* = 3). Unpaired *t* test was used to determine data significance. (**E, F**) IGV genome browser tracks representing the H3K27ac ChIP-seq peaks at the *Ptgs1* and *Flt1* loci in control (shGFP) and GPS2, SMRT and NCOR knockdown cells (*n* = 2). The peak changes within enhancer (E1, E2) and promoter (P) regions are highlighted in percentage. (**G–I**) MA plot showing the significant H3K27ac peak changes in shGPS2 (**G**), shSMRT (**H**) and shNCOR (**I**) RAW cells under basal conditions (*n* = 2). Significantly up-regulated and down-regulated peaks are highlighted in purple or blue. Data were re-analyzed from GSM4848501, GSM4848502, GSM4848505, GSM4848506. (**J**) IGV genome browser tracks representing the GPS2 ChIP-seq peaks at the *Ptgs1* locus in NCOR and SMRT knockdown cells (*n* = 2). (**K**) RT-qPCR analysis of *Ptgs1* expression upon GPS2/NCOR or GPS2/SMRT double depletion (*n* = 3). One-way ANOVA test was used for multiple comparisons. All data are represented as mean ± s.e.m. *P < 0.05, **P < 0.01, ***P < 0.001.

To determine whether GPS2 functions within the corepressor complex to regulate IL4 signaling, we included the GPS2-interacting core subunits NCOR (NCOR1) or SMRT (NCOR2) in our analysis. Re-analysis of our previous ChIP-seq data (37) revealed that GPS2, NCOR and SMRT colocalize in H3K27ac-positive chromatin regions (i.e. active enhancers and promoters) at the *Ptgs1* and *Mrc1* loci in RAW cells (Figure 2A and Supplementary Figure S2A). We also compared GPS2, NCOR, SMRT with IL4-induced H3K27ac regions (Supplementary Figure S2B). We found that over 95% of IL4 target H3K27ac regions are co-occupied by NCOR, SMRT and GPS2, consistent with their genome-wide co-occupancy. Among the IL4 target regions, depletion of GPS2 significantly enhanced the H3K27ac signals (Supplementary Figure S2D). We next studied the IL4 response separately in GPS2, NCOR and SMRT knockdown RAW cells. We selected 6 h for the IL4 treatment because IL4-induced *Ptgs1* mRNA expression in RAW cells and BMDMs peaked at this time point (Supplementary Figure S2C). Lentivirus shRNAs efficiently reduced GPS2, NCOR and SMRT both at the mRNA and protein levels (Supplementary Figure S2E-2H). Depletion of either GPS2, NCOR or SMRT increased basal and IL4-induced expression of *Ptgs1* (Figure 2B–D and Supplementary Figure S2H) and *Mrc1* (Supplementary Figure S2F). Consistent with this, H3K27ac levels at the *Ptgs1* promoter and enhancer were increased upon GPS2, NCOR or SMRT removal (Figure 2E), which was confirmed by ChIP-qPCR (Supplementary Figure S2I). Similar findings were observed at the *Mrc1* and *Flt1* gene loci (Figure 2F and Supplementary Figure S2J). Genome-wide analysis of H3K27ac changes in GPS2, NCOR and SMRT knockdown cells confirmed the coregulation of GPS2, NCOR and SMRT at IL4 target genes (Figure 2G–I). Noteworthy, *Ccl2* showed GPS2/SMRT selectivity, consistent with our previous findings. The coregulation of NCOR and SMRT with GPS2 in controlling *Ptgs1* expression was further confirmed by GPS2 ChIP-seq analysis in NCOR or SMRT knockdown cells, revealing strong reduction (shNCOR) or abolishment (shSMRT) of GPS2 binding at the *Ptgs1* locus (Figure 2J). Also, GPS2 depletion did not further increase *Ptgs1* expression in both NCOR- or SMRT-depleted RAW cells, suggesting that the two subunits play redundant but essential roles in recruiting GPS2 and assembling the chromatin-bound corepressor complex to control gene expression (Figure 2K). In sum, these data suggest that GPS2 acts within the corepressor core complex and requires both SMRT and, to a lesser extent, NCOR to exert its repressive function at IL4-regulated gene loci.

### Corepressor depletion increases chromatin accessibility at IL4 target gene loci

**Figure 3.**
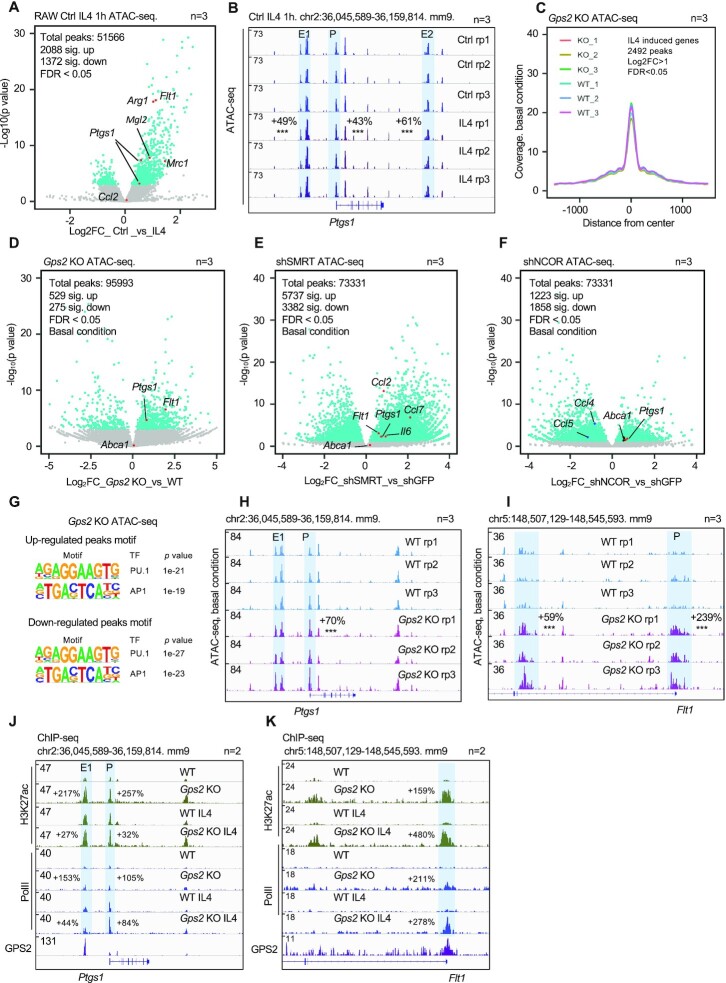
GPS2 restricts chromatin accessibility at IL4 target gene loci. (**A**) Volcano plot showing log_2_-fold changes of ATAC-seq peaks between control and IL4-treated RAW cells. The significantly up-regulated or down-regulated peaks are highlighted in blue. Peaks at marker gene loci are highlighted in red (*n* = 3). (**B**) IGV genome browser tracks of ATAC-seq at the *Ptgs1* locus in control and IL4-treated RAW cells. (**C**) Peak coverage representing genome-wide ATAC-seq changes in WT versus *Gps2* KO RAW cells under basal condition (*n* = 3). (D–F) Volcano plot showing log_2_-fold changes of ATAC-seq peaks between depletion of GPS2 (**D**), SMRT (**E**) and NCOR (**F**) vs. control cells. The significantly up-regulated or down-regulated peaks are highlighted in blue. Peaks at marker gene loci are highlighted in red. (**G**) Motif analysis of the significantly up- and down-regulated ATAC-seq peaks in *Gps2* KO versus WT RAW cells. (**H-I**) IGV genome browser tracks representing the ATAC-seq peaks in WT and *Gps2* KO RAW cells at the *Ptgs1* (**H**) and *Flt1* (**I**) loci. Enhancers and promoters are highlighted. (**J, K**) IGV genome browser tracks representing the H3K27ac mark and Pol II recruitment at the *Ptgs1* and *Flt1* loci in WT and *Gps2* KO cells under basal condition versus IL4 treatment. Unpaired *t-*test was used to determine data significance. All data are represented as mean ± s.e.m. *P < 0.05, **P < 0.01, ***P < 0.001.

Because GPS2, along with SMRT and NCOR, might create a repressive chromatin environment influencing chromatin accessibility, we performed ATAC-seq upon IL4-treatment or individual depletion of the complex subunits. We found that IL4 treatment specifically increased chromatin accessibility at IL4 target genes such as *Ptgs1, Mrc1, Flt1* but not genome-widely (Figure 3A and B, Supplementary Figure S3A–C). Similarly, genome-wide changes upon GPS2 depletion were marginal (Figure 3C). However, there were IL4 target gene-specific effects of GPS2 depletion on chromatin accessibility, consistent with the gene expression data. For example, ATAC-seq signals at the *Ptgs1* locus were increased in shGPS2, shSMRT and shNCOR RAW cells, while no change upon GPS2 depletion was seen at previously identified inflammatory chemokine *Ccl2* sites (Figure 3D–F). AP1 and PU.1 were the top TF motifs enriched in both elevated and reduced ATAC-seq regions upon deletion of GPS2 (Figure 3G), SMRT or NCOR (Supplementary Figure S3D and E). In IL4-treated cells, the up-regulated ATAC-seq peaks were additionally enriched with STAT6 motifs, while CEBP motifs were enriched in the down-regulated peaks (Supplementary Figure S3F). In IL4-induced regions, chromatin accessibility was associated with STAT6 recruitment (Supplementary Figure S3G). Comparing the ATAC-seq peak sizes upon depletion of SMRT, NCOR or GPS2 revealed that NCOR and SMRT-specific peaks were higher and narrower than GPS2, suggesting stronger and more specific binding (Supplementary Figure S3H). ATAC-seq genome browser shots of *Ptgs1* and *Flt1*, both annotated to be significantly altered, revealed increased chromatin accessibility at their promoter and enhancer regions (Figure 3H and I, Supplementary Figure S3I and J). Consistently, both H3K27ac and RNA polymerase II (Pol II) at the promoter (*Ptgs1* and *Flt1*) and enhancer (*Ptgs1*) regions were elevated in both basal and IL4 activation conditions in *Gps2* KO cells (Figure 3J and K). These data uncover a hitherto unknown role of the GPS2-containing corepressor complex in propagating a less-accessible chromatin environment at enhancers and promoters, likely contributing to its repressive function.

### GPS2 regulation of the IL4 response depends on STAT6, but repression of IL4 target enhancers does not

**Figure 4.**
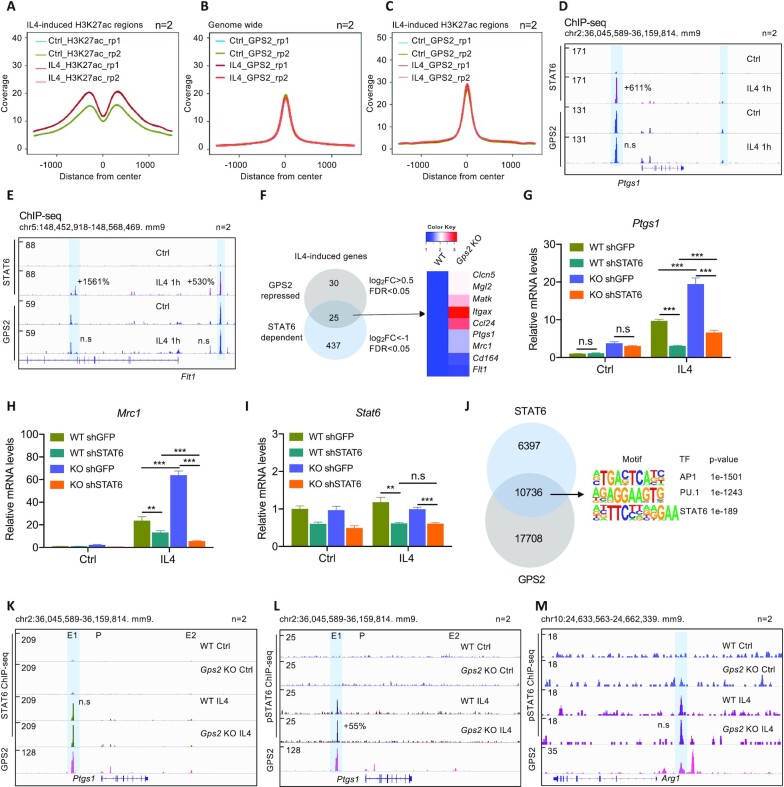
Increased IL4 response upon GPS2 depletion is dependent on STAT6. (A–C) Peak coverage plots of H3K27ac (**A**), GPS2 genome-wide (**B**) or GPS2 at H3K27ac-changed regions (**C**) upon control versus IL4 treatment in RAW cells. (**D, E**) IGV genome browser tracks representing STAT6 and GPS2 occupancy at the *Ptgs1* and *Flt1* loci in IL4-treated RAW cells (*n* = 2); (**F**) Venn diagram showing the overlap of STAT6-dependent and GPS2-repressed genes (left panel), along with the heatmap showing the overlapped gene list (right panel). (**G–I**) RT-qPCR analysis of *Ptgs1*, *Mrc1* and *Stat6* expression in STAT6-depleted WT and *Gps2* KO RAW cells (*n* = 3). (**J**) Venn diagram showing STAT6- and GPS2-occupied regions and motif enrichment in the common peaks. (K–M) IGV genome browser tracks representing recruitment of STAT6 and GPS2 (**K**) or p-STAT6 and GPS2 (L, M) at the *Ptgs1* (**L**) and *Arg1* (**M**) loci in WT versus *Gps2* KO cells under basal condition versus IL4 treatment (*n* = 2). All data are represented as mean ± s.e.m. *P < 0.05, **P < 0.01, ***P < 0.001.

To further dissect the regulatory mechanisms of GPS2 on IL4 signaling in macrophages, we tested whether IL4 could induce GPS2 recruitment changes at its target gene loci. IL4 treatment led to a specific cluster of H3K27ac up-regulation in RAW cells (Figure 4A). Interestingly, despite significant increase of H3K27ac in GPS2, NCOR or SMRT versus IL4 target H3K27ac co-binding regions (Supplementary Figure S4A–C), and STAT6 (in NCOR and SMRT KD cells in response to IL4, Supplementary Figure S4B and C), the overall ATAC-seq peaks remain unchanged in all groups (Supplementary Figure S4A–C). These data suggest that the changed chromatin accessibility is limited to a subset of IL4 target genes/loci and might generally not be essential for altered chromatin and transcriptional activities upon corepressor depletion. The TF motif analysis in GPS2, NCOR and SMRT-repressed IL4 target H3K27ac regions revealed enrichment of AP1, PU.1 and STAT6 (Supplementary Figure S4D). Unexpectedly, GPS2 binding was not affected by IL4 both globally (Figure 4B) and at IL4 target loci marked by increased H3K27ac (Figure 4C). In contrast, LPS induced a genome-wide release of GPS2 from its binding sites (Supplementary Figure S5C), consistent with our previous findings (37). The genome-wide analysis was confirmed at the individual IL4 target gene loci *Ptgs1* and *Flt1* (Figure 4D and E) and by ChIP-qPCR (Supplementary Figure S5A and B).

Because STAT6 is the major TF crucial for IL4 pathways (14,15,18), we explored whether STAT6 was required for GPS2 repression. IL4 up-regulated PTGS1 protein levels in a time-dependent manner paralleled with STAT6 phosphorylation (p-STAT6) dynamics in RAW cells (Supplementary Figure S5D). Consistently, STAT6 recruitment at both *Ptgs1* and *Flt1* loci was induced by IL4 (Figure 4D and E). Transcriptome comparison between GPS2-repressed and STAT6-induced IL4 signature genes confirmed *Ptgs1, Mrc1* and *Flt1* as their common targets in RAW cells (Figure 4F). Analysis of public RNA-seq (15) revealed that this regulation was conserved in BMDMs, as IL4-induced expression of *Ptgs1*, *Mrc1* and *Flt1* was abrogated in *Stat6* KO BMDMs, as compared to WT BMDMs (Supplementary Figure S5E). Knockdown of STAT6 using lentivirus shRNA significantly reduced *Ptgs1* and *Mrc1* expression upon IL4 treatment in both WT and *Gps2* KO RAW cells, and the GPS2 regulation on both genes was largely attenuated (Figure 4G–I).

We also addressed the role of PPARγ, known to cooperate with STAT6 in IL4 pathways (53). PPARγ recruitment at the *Ptgs1* locus was induced by IL4 in RAW cells (Supplementary Figure S5F). PPARγ was functional at other genes as treatment with rosiglitazone, a PPARγ agonist, increased expression of *Ap2* (*Fabp4*, a classic PPARγ target gene) similarly as IL4 (Supplementary Figure S5G). However, rosiglitazone did not increase *Ptgs1* expression (Supplementary Figure S5H), and knockdown of PPARγ using lentivirus shRNA did not reduce IL4-induced *Ptgs1* expression (Supplementary Figure S5I and S5J). This contrasts the requirement of STAT6 at genes which are repressed by GPS2 and makes the involvement of PPARγ unlikely.

We then continued to explore whether STAT6 and GPS2 cooperation in the IL4 pathway was through direct interactions. We performed ChIP-seq and compared the genome-wide peaks of GPS2 and STAT6 with the result that more than 30% of all peaks overlapped, enriched with mainly AP1, PU.1 and STAT6 motifs (Figure 4J). Both total STAT6 and phospho-STAT6 (p-STAT6) levels were unchanged in *Gps2* KO RAW cells (Supplementary Figure S5K). Likewise, STAT6 knockdown did not affect GPS2 levels (Supplementary Figure S5L). Co-immunoprecipitation indicated that GPS2 does not physically interact STAT6, while interactions of GPS2 with SMRT was readily observed under similar conditions (Supplementary Figure S5M). Although IL4 significantly enhanced STAT6 binding both genome-widely and in the elevated H3K27ac regions (Supplementary Figure S5N and O), global STAT6 recruitment in WT and *Gps2* KO cells showed minor differences (Supplementary Figure S5P), which was also observed at the *Ptgs1* locus (Figure 4K). Likewise, GPS2 recruitment was not affected by STAT6 depletion, as compared to control cells (Supplementary Figure S5Q and R). Interestingly, although GPS2 depletion had no effects on global p-STAT6 levels (Supplementary Figure S5K), the p-STAT6 recruitment at the *Ptgs1* locus seemed increased by more than 50% (Figure 4L). This seems to correlate with transcriptional changes, as no change was seen at the *Arg1* gene locus (Figure 4M), consistent with *Arg1* not being up-regulated by GPS2 depletion (Figure 1C). Overall, these data suggest that GPS2 represses IL4-dependent STAT6 activation not through direct interaction or by regulating STAT6 chromatin access at enhancers. Instead, GPS2 likely controls other steps of STAT6 activation, possibly by modulating the action of coactivators and chromatin modifiers.

### GPS2 antagonizes KDM1A in IL4 target gene activation and histone H3K9 demethylation

**Figure 5.**
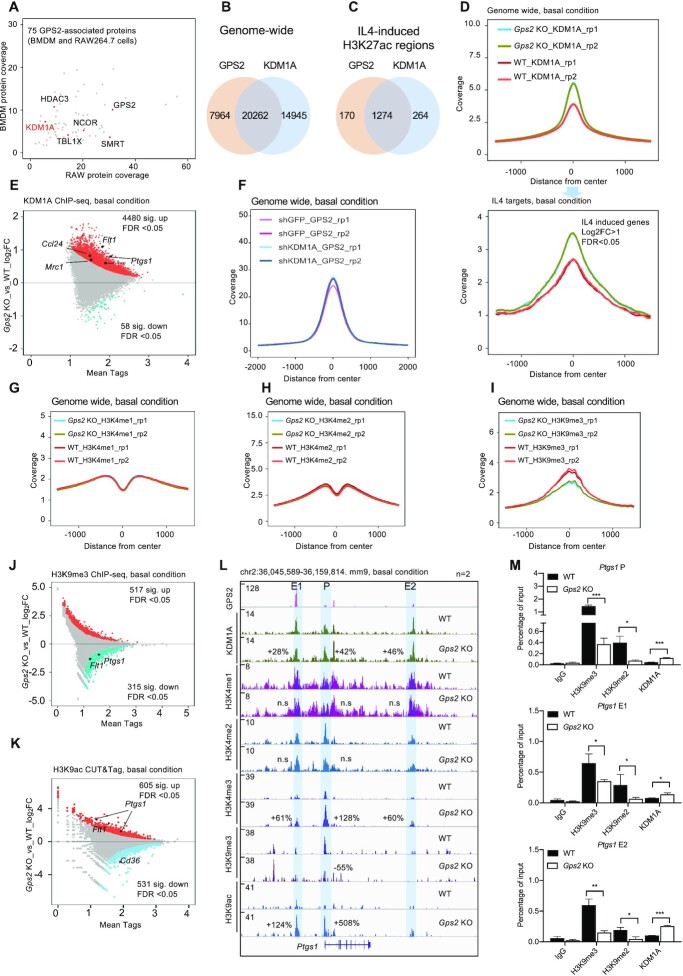
GPS2 antagonizes KDM1A to restrict H3K9me2/3 demethylation at IL4 target gene loci. (**A**) Scatter plot representing chromatin-associated GPS2 interaction partners from both RAW cells (X-axis) and BMDMs (y-axis). Protein coverage rate was used for the plot. All core subunits of the corepressor complex are labelled and KDM1A is highlighted in red. (**B, C**) Venn diagram showing the overlap of GPS2 and KDM1A ChIP-seq peaks in both genome-wide and IL4 response regions. (**D**) KDM1A ChIP-seq peak coverage genome-widely (upper panel) and at IL4-inducible gene loci (lower panel) in WT versus *Gps2* KO RAW cells under basal condition (*n* = 2); (**E**) MA plot showing the KDM1A ChIP-seq peak changes in *Gps2* KO versus WT RAW cells under basal condition (*n*= 2). Significantly up-regulated and down-regulated peaks are highlighted in red or blue. *Ptgs1, Mrc1, Flt1* and *Ccl24* peaks are labelled. (**F**) GPS2 peak coverage upon KDM1A depletion in basal condition (*n* = 2). (G–I) Genome-wide ChIP-seq peak coverage of H3K4me1 (**G**), H3K4me2 (**H**) and H3K9me3 (**I**) in *Gps2* KO versus WT RAW cells (*n* = 2). (**J, K**) MA plots showing H3K9me3 and H3K9ac ChIP-seq/CUT&Tag peak changes in *Gps2* KO versus WT RAW cells under basal condition (*n* = 2). Significantly up-regulated and down-regulated peaks are highlighted. *Ptgs1* and *Flt1* peaks are labelled in the plot. (**L**) IGV genome browser tracks representing KDM1A occupancy and levels of the indicated histone modifications at the *Ptgs1* locus in WT and *Gps2* KO cells under basal condition. Significantly changed regions are highlighted. GPS2 ChIP-seq tracks were used to mark the promoter (P) and enhancers (E1, E2). (**M**) ChIP-qPCR analysis of KDM1A, H3K9me2, and H3K9me3 levels at the *Ptgs1* promoter (P) and enhancers (E1, E2) in WT versus *Gps2* KO cells (*n* = 3). Unpaired *t-*test was used to determine data significance. All data are represented as mean ± s.e.m. *P < 0.05, **P < 0.01, ***P < 0.001.

To find such candidate coactivators and chromatin modifiers, we explored the GPS2-associated chromatin interactome using rapid immunoprecipitation mass spectrometry (42) (RIME, ChIP-MS) in RAW cells and BMDMs (Figure 5A). In addition to all corepressor complex core subunits serving as a positive control, we identified the lysine-specific demethylase 1A (KDM1A, also known as LSD1) (54) in the GPS2-specific immunoprecipitates from both macrophage cell types (Figure 5A). Although KDM1A has been extensively studied in multiple cell types and disease contexts (55–59), only few recent studies have explored its role in M1 macrophages (60–63), and its specific function in IL4 signaling and M2 macrophages has not yet been addressed. We thus performed ChIP-seq and compared the cistromes of GPS2, KDM1A and STAT6. Consistently, all three proteins were recruited to the *Ptgs1* promoter and enhancers, along with PU.1 and JUNB (AP1) (Supplementary Figure S6A). Genome-widely, more than 70% of GPS2 peaks overlapped with around 60% of KDM1A peaks (Figure 5B), and this overlap was even higher at IL4-responsive H3K27ac regions (Figure 5C). To address the role of GPS2 in KDM1A action, we performed KDM1A ChIP-seq in WT versus GPS2-depleted RAW cells and found that GPS2 depletion triggered enhanced KDM1A recruitment both genome-widely and at IL4 target gene loci including *Ptgs1*, *Mrc1* and *Flt1* (Figure 5D and E). In contrast, KDM1A knockdown did not affect GPS2 binding in the RAW cells (Figure 5F). Consistent with the increase of chromatin-bound KDM1A and its coactivator function, GPS2 depletion by either CRISPR (*Gps2* KO) or shRNA (shGPS2) knockdown resulted in increased H3K4me3 (active promoter mark) and H3K27ac (active enhancer and promoter mark) both genome-widely and at IL4 target gene loci (Supplementary Figure S6B-S6C). We next determined whether increased KDM1A upon GPS2 depletion caused changes of several histone H3K4 and H3K9 methylation marks known to be direct substrates (H3K4me1, H3K4me2, H3K9me2) (54,64) or indirectly affected by KDM1A (H3K9me3) (65,66). ChIP-seq demonstrated that genome-wide H3K4me1 and H3K4me2 levels were not affected by GPS2 depletion (Figure 5G-H and Supplementary Figure S6D-S6F). However, H3K9me3 levels significantly decreased both genome-widely and at IL4 target genes, consistent with H3K9ac increases at the same loci (Figure 5I-L). The direct substrate of KDM1A, H3K9me2 was evaluated using ChIP-qPCR (as we tested several commercial H3K9me2 antibodies to be insufficient for ChIP-seq in the RAW cell line, *data not shown*) at the *Ptgs1* locus, which revealed a reduction of H3K9me2, comparable to H3K9me3, upon GPS2 depletion (Figure 5M).

We additionally analyzed the GPS2-repressed and IL4-induced genes/loci with or without STAT6 binding sites in basal and IL4 condition (Supplementary Figure S6G). Although chromatin accessibility and STAT6 binding were not altered upon GPS2 depletion in the GPS2 target IL4 genes, both KDM1A recruitment and H3K27ac were enhanced in STAT6 positive regions. These results were further confirmed at IL4 target genes including *Ptgs1*, *Flt1* and *Mgl2* (Figure 5L, Supplementary Figure S7A).

STAT6 largely colocalized with KDM1A and the corepressor complex (GPS2, NCOR and SMRT) (Supplementary Figure S7B–D). Similar to GPS2 depletion, NCOR and SMRT depletion led to increased KDM1A recruitment at the *Ptgs1* locus (Supplementary Figure S7E), supporting them to operate together in the complex.

Finally, we analyzed the relative mRNA expression of KDM1A, along with other relevant KDMs implicated in H3K9me2/3 demethylation, from RNA-seq in WT versus GPS2-depleted cells, which revealed no changes (Supplementary Figure S7F). This suggests that increased KDM1A recruitment and decreased H3K9 methylation upon GPS2 depletion was not due to elevated expression of these KDMs (Supplementary Figure S7F). GPS2 and KDM1A protein levels were not altered by IL4 treatment (Supplementary Figure S7G). Further, upon IL4 activation KDM1A remained bound to the *Ptgs1* promoter and enhancer, where the H3K27ac and H3K4me3 activity marks were increased (Supplementary Figure S7H). Overall, these results point at a mechanism by which GPS2 represses IL4 target gene activation in part by preventing chromatin access of KDM1A, thereby suppressing the coactivator function of KDM1A and maintaining repressive H3K9me2/3 chromatin states within IL4 target gene loci.

### KDM1A acts as a coactivator and H3K9 demethylase in the IL4 pathway

**Figure 6.**
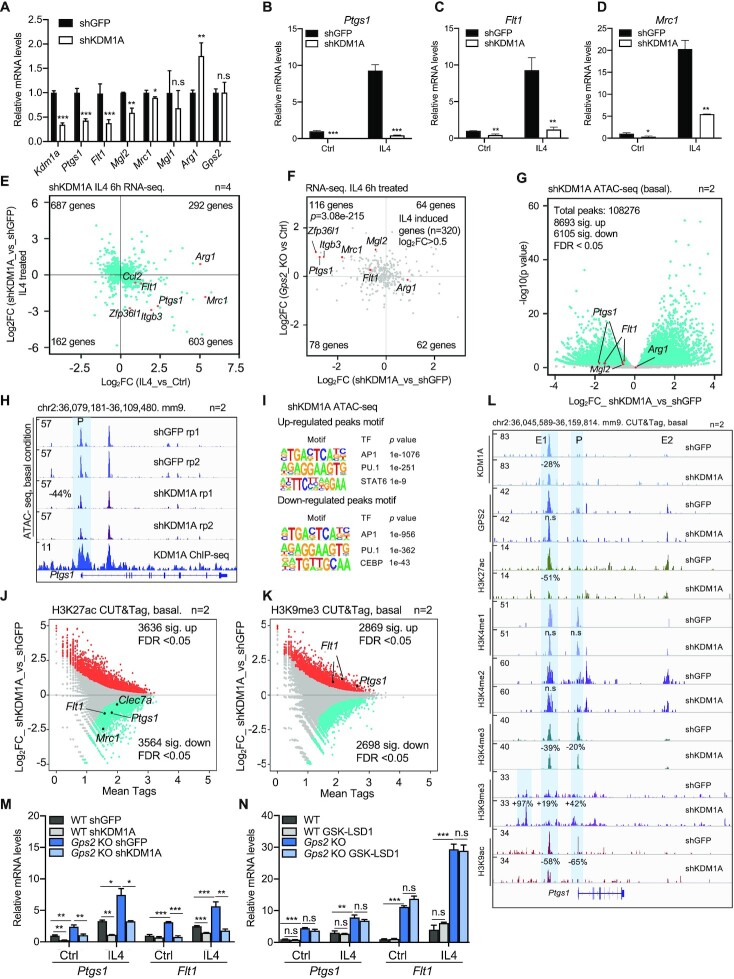
(**A**) RT-qPCR analysis of IL4 target gene expression in lentivirus shGFP versus shKDM1A RAW cells under control condition (*n* = 3). (B–D) RT-qPCR analysis of *Ptgs1* (**B**), *Flt1* (**C**), *Mrc1* (**D**) gene expression in shGFP versus shKDM1A RAW cells under upon IL4 treatment (*n* = 3). (**E**) Scatter plot showing the comparison of transcriptome signatures between KDM1A knockdown and IL4 treatment in BMDMs. The X-axis represents the gene log_2_-fold changes in IL4 versus control BMDMs, and the Y-axis represents the gene log_2_-fold changes in IL4-treated shKDM1A knockdown versus shGFP control BMDMs. All significantly changed genes are presented in each quadrant. (**F**) Scatter plot comparing GPS2- and KDM1A-regulated genes in the IL4 pathway. IL4-induced genes were pre-selected using log_2_-fold change > 0.5 as a ratio. All changed genes are presented in each quadrant. GPS2-repressed and KDM1A-activated genes were used for the hypergeometric test (phyper), which used the total number of mouse genes (25059) as background. The test *P-*value is highlighted in the first quadrant. The associated genes are further highlighted. (**G**) Volcano plot showing the genome-wide ATAC-seq peak changes in shKDM1A RAW cells. Peak changes within the indicated IL4 target gene loci are highlighted in red. (**H**) IGV genome browser tracks of ATAC-seq at the *Ptgs1* locus in shGFP and shKDM1A RAW cells under basal condition (*n* = 2). The *Ptgs1* promoter (P) region is highlighted. KDM1A ChIP-seq is presented in the lower panel. (**I**) TF motif analysis of the significantly up- and down-regulated ATAC-seq peaks in shKDM1A versus shGFP RAW cells. (J, K) MA plots showing the H3K27ac (**J**) and H3K9me3 (**K**) CUT&Tag peak changes in shKDM1A versus shGFP cells under basal condition (*n* = 2). Significantly up- and down-regulated peaks are highlighted in red or blue in each plot. (**L**) IGV genome browser tracks representing the CUT&Tag peaks of KDM1A, GPS2 along with the indicated histone marks at the *Ptgs1* locus in shKDM1A versus shGFP RAW cells under basal condition (*n* = 2). Significantly changed peaks are highlighted in blue. (**M**) RT-qPCR analysis of *Ptgs1* and *Flt1* expression in control and IL4 condition in WT versus GPS2 and KDM1A single versus double depleted RAW cells (*n* = 3). (**N**) RT-qPCR analysis of *Ptgs1* and *Flt1* expression in control and IL4 condition upon treatment with KDM1A inhibitor GSK-LSD1 in WT versus GPS2-depleted RAW cells (*n* = 3). Unpaired *t-*test was used to determine data significance. All data are represented as mean ± s.e.m. *P < 0.05, **P < 0.01, ***P < 0.001.

To further dissect the mechanisms underlying GPS2 and KDM1A antagonism in IL4 signaling, we analyzed the consequences of KDM1A depletion, comparable to above-described analysis of GPS2 depletion (Figure 1-3). KDM1A expression decreased by more than 60% upon lentivirus shRNA-mediated knockdown in RAW cells (Figure 6A and Supplementary Figure S8A). IL4 target genes such as *Ptgs1*, *Mrc1* and *Flt1 (*but not *Arg1)* were downregulated upon KDM1A depletion in un-stimulated cells (Figure 6A). Strikingly, IL4 induction of these genes was strongly reduced upon KDM1A removal (Figure 6B–D). These data were confirmed at the genome-wide level by RNA-seq analysis in shGFP versus shKDM1A cells treated with IL4 (Figure 6E). Comparison to GPS2-depleted cells (Figure 6F) revealed that many genes that were repressed by GPS2 were also activated by KDM1A, supporting their antagonistic action in IL4 target gene expression. We then performed ATAC-seq with the result that KDM1A depletion, while not causing genome-wide changes (Figure 6G), led to reduced chromatin accessibility at IL4 target gene loci, including *Ptgs1, Mrc1* and *Flt1* (Figure 6H, Supplementary Figure S8B and C). The top TF motifs enriched in up- or down-regulated ATAC-seq peaks upon KDM1A knockdown were AP1 and PU.1, while STAT6 was linked to up-regulation and CEBP to down-regulation (Figure 6I). We when performed CUT&Tag and found that KDM1A depletion, while not significantly changing GPS2 recruitment to IL4 target loci (Supplementary Figure S8D), decreased H3K27ac and H3K4me3, and notably increased H3K9me3 at the *Ptgs1*, *Mrc1* and *Flt1* loci (Figure 6J–L and Supplementary Figure S8G, H), consistent with KDM1A acting as a coactivator opposing GPS2. In contrast, H3K4me1 and H3K4me2 were not changed upon KDM1A depletion at the *Ptgs1* locus, consistent with no change upon GPS2 depletion (Figure 6L, Supplementary Figure S8E and F). The changes observed by CUT&Tag data were validated using ChIP-qPCR at the *Ptgs1* promoter (Supplementary Figure S8I). Intriguingly, western blot analysis revealed a robust up-regulation of total H3K9me2 levels, accompanied by slight upregulation of total H3K9me3 levels, in KDM1A-depleted RAW cells (Supplementary Figure S8J). Moreover, while KDM1A depletion had no effects on IL4-induced STAT6 expression and phosphorylation (Supplementary Figure S8K), STAT6 chromatin recruitment was reduced genome-widely (Supplementary Figure S8L and M) and at IL4 target gene loci, as shown for *Ptgs1* and *Flt1* (Supplementary Figure S8N). In contrast, GPS2 recruitment was not changed upon KDM1A depletion. One possible interpretation of these data is that KDM1A depletion maintains a repressive chromatin environment, marked by H3K9me2/3, that counteracts IL4-induced STAT6 recruitment to the enhancers and promoters of IL4-target gene loci.

Interestingly, KDM1A seems required for the de-repression of *Ptgs1* and *Flt1* transcription upon GPS2 depletion in both basal and IL4 condition, as double depletion of GPS2 and KDM1A abrogated the increased expression of these gene by GPS2 depletion alone (Figure 6M). However, in contrast to the gene expression changes upon KDM1A depletion (Figure 6A-D, M), the KDM1A inhibitor GSK-LSD1 did not affect the expression of *Ptgs1* and *Flt1* (Figure 6N), despite it functioned by increasing both the total and *Ptgs1* locus-specific H3K4me1/2 levels without changing H3K27ac (Figure 6N, Supplementary Figure S6H and S6I. These data suggest that the enzymatic KDM1A activity, while required for H3K4me1/2 de-methylation, is not required for the coactivator function of KDM1A, at least at a subset of major IL4/GPS2 target genes.

### PU.1 coordinates chromatin recruitment of GPS2 and KDM1A at IL4 target loci

**Figure 7.**
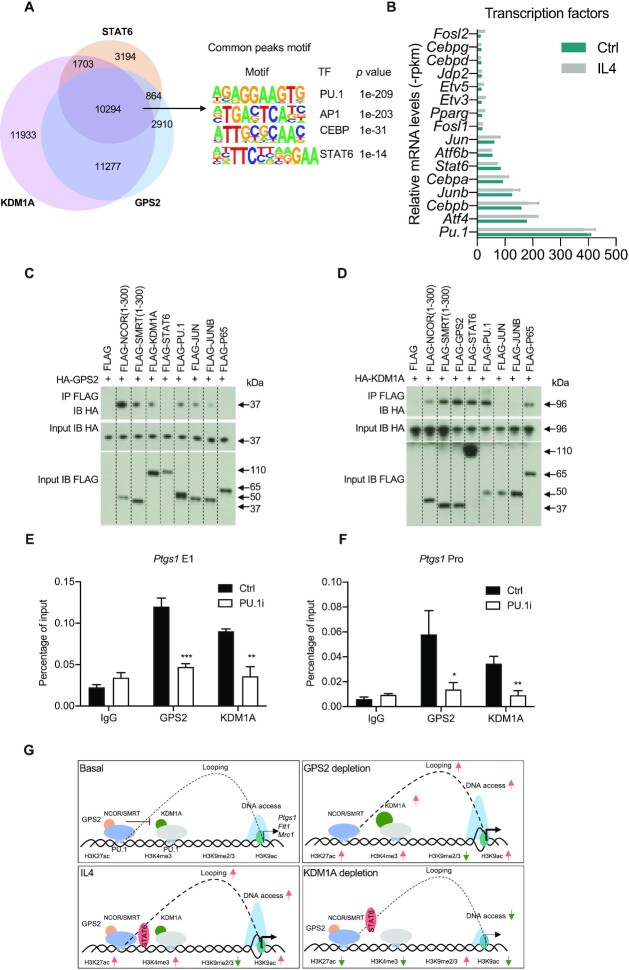
(**A**) Venn diagram showing the overlap of GPS2, STAT6, and KDM1A occupancy (obtained by ChIP-seq) in RAW cells (left panel), along with the enriched TF motifs in the common peaks. (**B**) RNA-seq tag counts (-rpkm) representing the relative TF gene expression in RAW cells upon 6 h IL4 treatment versus control. (**C**) Co-immunoprecipitation of HA-GPS2 with the indicated FLAG-tagged TFs and coregulators in HEK293 cells. Previously reported interactions with JUN, NCOR and SMRT served as positive controls (36) (**D**) Co-immunoprecipitation of HA-KDM1A with the indicated FLAG-tagged TFs and coregulators in HEK293 cells. Previously reported interaction with p65 served as positive control (74). (**E, F**) ChIP-qPCR of GPS2 and KDM1A at *Ptgs1* enhancer (E1) and promoter region (Pro) upon PU.1 inhibition (PU.1i) in RAW cells (*n* = 3). (**G**) Model of GPS2-KDM1A antagonism in regulating IL4 target gene expression during M2 macrophage activation. Unpaired *t* test was used to determine data significance. All data are represented as mean ± s.e.m. *P < 0.05, **P < 0.01, ***P < 0.001.

As GPS2-KDM1A antagonism likely involves the binding to specific TFs, we aimed to identify candidates using the following experiments. We first performed TF motif analysis of GPS2, NCOR, SMRT, KDM1A and STAT6 cistromes in RAW cells (Figure 7A and Supplementary Figure S9A). GPS2 shares more than 90% binding regions with over 60% of KDM1A and STAT6 peaks, and PU.1 and AP1 appear as the top TF motifs (Figure 7A). These TF motifs rank top also in the shared peaks between GPS2, NCOR and SMRT, marking the corepressor complex cistrome (Supplementary Figure 9A). Consistent with this, a coverage plot revealed that GPS2, NCOR, SMRT and KDM1A share binding patterns with PU.1 and JUNB (AP1) (Supplementary Figure S9B). Independent support was provided by TF activity analysis based on the transcriptome signature changes in GPS2-depleted or KDM1A-depleted macrophages. Both depletions resulted in increased activity of PU.1 and of several members of the AP1 family (Supplementary Figure S9C and D). Supporting the functional relevance of above TFs, we next determined the relative TF mRNA expression in BMDMs and RAW cells. This revealed that PU.1 and AP1 family members (e.g. JUNB, JUN) were amongst the highest expressed TFs of interest, and that their expression was not altered upon IL4 treatment (Figure 7B). We additionally found that the expression of these and other TF family members was not affected by depletion of either GPS2 or KDM1A (Supplementary Figure S9E and F). We finally investigated interactions of highly expressed inflammatory TFs with GPS2 and KDM1A using co-immunoprecipitation. While GPS2 interacted with PU.1, JUN and JUNB (Figure 7C), KDM1A interacted with PU.1, STAT6 and p65 (Figure 7D). The functional relevance of the shared PU.1 interactions was supported by inhibition of PU.1 DNA binding, which caused dissociation of GPS2 and KDM1A from chromatin at the *Ptgs1* enhancer and promoter regions (Figure 7E and F). We conclude that PU.1 could be one of the major TFs that mediate the chromatin recruitment and antagonism of GPS2 and KDM1A at IL4-regulated macrophage enhancers. A working model integrating all findings of this study is presented in Figure 7G.

## DISCUSSION

Our study uncovered hitherto unknown epigenetic mechanisms by which the antagonistic action of a major corepressor complex and a major histone demethylase control IL4-dependent macrophage activation and maintain a repressive basal state prior to IL4 activation. There are at least three aspects supporting these mechanisms, discussed further below and highlighted in our model (Figure 7G).

1. The corepressor core subunit GPS2 represses both basal and IL4-induced gene expression (as judged by transcriptome analysis via RNA-seq) by occupying enhancers (as judged by cistrome analysis via ChIP-seq, CUT&Tag) and by limiting enhancer-promoter looping (as judged by 4C-seq) within the TADs of IL4 target gene loci. At some enhancers GPS2 seems in addition to restrict chromatin access (as judged by ATAC-seq). In all of above steps, GPS2 seems to cooperate with SMRT and NCOR, suggesting GPS2 to function as an integral subunit of the chromatin-associated corepressor complex at IL4 target gene loci.
2. The macrophage GPS2 corepressor complex associates with the histone demethylase KDM1A at chromatin, as we identified it using ChIP-MS (RIME) independently from both BMDMs and RAW cells. Functional analysis revealed that GPS2 (along with SMRT, NCOR) and KDM1A co-occupy IL4 target gene enhancers but function antagonistic at several levels. The corepressor complex restricts chromatin access of KDM1A at enhancers (i.e. corepressor depletion increases KDM1A chromatin occupancy) and antagonizes the demethylase-independent coactivator function of KDM1A (as judged from inhibitor treatment) in the transcriptional activation of IL4 target genes.
3. Activation of STAT6, the major IL4-induced TF which upon IL4 signaling co-occupies enhancers along with the GPS2 corepressor complex and KDM1A, is affected at different levels by either GPS2 depletion (STAT6 phosphorylation) or KDM1A depletion (reduced chromatin access). STAT6 is however unlikely to be the direct target TF for the corepressor-coactivator antagonism at chromatin. Since GPS2 represses and antagonizes KDM1A at IL4 target genes even in the absence of IL4 signaling, other TFs are likely required for recruiting the corepressor complex and KDM1A to chromatin, with PU.1 being a strong candidate.

Our current study extents previous work and further emphasizes the dual role of the GPS2-containing corepressor complex, and its functional sub-complexes, in regulating both pro- and anti-inflammatory responses in macrophages. Because GPS2 expression, and thereby corepressor complex function, in tissue macrophages is altered in immuno-metabolic disease contexts in humans (20,36), our study should stimulate the further investigation of IL4 signaling in human macrophage subtypes. The comparison of GPS2 corepressor complex action in M1 versus M2 activation emphasizes that the function of coregulators in macrophages is highly signal-specific, most likely due to the different sets of TFs that respond to these signals. The physiological consequence of altered GPS2 expression is therefore dependent on the combination of different stimuli typically occurring *in vivo*. While GPS2 acts largely anti-inflammatory by inhibiting M1-type metabolic inflammation under conditions of obesity and diabetes (20,36,37), it might act rather pro-inflammatory in a M2-type micro-environment, for example in macrophages involved in wound healing (6) or in tumor-associated macrophages involved in metastasis (9). All these aspects could be of relevance for the here identified GPS2-KDM1A antagonism in complex disease environments.

Our study provides further insights into the fundamental question of whether GPS2 acts within or without the corepressor complex, and how it cooperates with the other core subunits NCOR or SMRT to control macrophage function (20). Whereas our previous studies have revealed that GPS2 cooperates with SMRT but not NCOR to control pro-inflammatory gene transcription in M1-type macrophages (35–37), we show here that repression of IL4-regulated anti-inflammatory gene transcription in M2-type macrophages requires all three core subunits. These data further support the gene- and signal-selectivity of corepressor sub-complexes in macrophages. Intriguingly, while NCOR, SMRT and GPS2 colocalize at all IL4 target genes, depletion of SMRT abolished, and depletion of NCOR reduced GPS2 binding, strongly supporting that GPS2 requires these subunits to bind chromatin and to modulate transcription. Possibly, deletion of either subunit destabilizes interactions of the corepressor complex with TFs, histone modifiers such as KDMs, and histone tails to alter the local TAD-intrinsic 3D chromatin structure and accessibility along with the formation of enhancer–promoter loops to stimulate transcription, as shown here for IL4 signaling. Sub-complexes may still operate even in the M2 pathway, likely due to binding preferences for different TFs and TF combinations at IL4-regulated enhancers. This may explain why expression of *Arg1*, a typical IL4/STAT6 target in mouse macrophages, seems not repressed by GPS2 in our study, while it was repressed by NCOR and HDAC3 in BMDMs (32–34).

Our study addresses the role of macrophage TFs in GPS2-KDM1A antagonism at IL4-inducible enhancers and target gene loci. The outcome suggests that STAT6, although required for IL4-induced gene expression and its activation is modulated by GPS2 and KDM1A, it is not a direct target for these coregulators. Instead, other TFs such as PU.1 are likely responsible for recruiting the GPS2 corepressor complex and KDM1A to chromatin. Major arguments are that antagonisms occurs in unstimulated macrophages in the absence of STAT6/IL4 signaling, where the GPS2 corepressor complex bookmarks the enhancers of IL4 target genes. Competitive TF interactions of KDM1A and GPS2 may explain how depletion of GPS2 increases the enhancer recruitment of KDM1A, which possibly cooperates with CBP/p300 to increase H3K27ac, eRNA transcription and enhancer-promoter communication. Thus, the integration of KDM1A extents the corepressor-dependent mechanisms of M1 macrophage enhancer activation discovered in our previous study (37) and suggests such mechanisms to operate both in IL4 (M2) and LPS (M1) contexts. Interestingly, previous work has revealed that HDAC3 is involved in repressing the IL4 pathway in BMDMs (33). Since HDAC3 needs to interact with NCOR/SMRT to access chromatin (20,28), and since we show that NCOR/SMRT depletion has similar effects as GPS2 depletion in the IL4 pathway, HDAC3 is a likely component of the KDM1A antagonism executed by the corepressor complex. Moreover, the results of our study do not conflict with a previous report that NCOR and HDAC3 associate with STAT6 at a subset of IL4-repressed enhancers (15). Docking mechanisms independent of direct STAT6 interaction, likely mediated by LDTFs including PU.1, and repression mechanisms independent of GPS2 and SMRT, may account for this peculiar STAT6-dependent gene repression.

Our study advances the understanding of functions and target range of KDM1A, the first identified and probably best-studied KDM, also known as lysine-specific demethylase 1 (LSD1) (54). Although KDM1A has been extensively studied in multiple cell types and disease contexts (55–59), its specific function in IL4 signaling and M2 macrophages has not yet been addressed and only few recent studies have explored its role in M1 macrophages (60–63). Our data indicate that KDM1A opposes the GPS2 corepressor complex by acting a coactivator of IL4 signaling, as its depletion abolishes IL4-induced gene expression and increases H3K9me2/3 levels, both at the genome-wide level and within the specific IL4 target gene loci. Notably, the classic KDM1A substrates H3K4me1/2 linked to its repressive function were not changed upon KDM1A depletion. This suggests that the coactivator function of KDM1A in the macrophage context may involve the modulation of repressive H3K9me2/3 marked chromatin regions that exist even within active euchromatin regions defined by TADs (67). This also suggests that KDM1A operates different than in other cellular and signaling contexts, likely independently of the CoREST/HDAC1/2 complexes (63,68–70). The detailed mechanisms regarding the selective regulation of KDM1A on H3K4 or H3K9 demethylation in different contexts remains unclear. It is possible that the repressive function of KDM1A in other cell types is dependent on interacting TFs such as the androgen receptor to facilitate its H3K4 demethylase activities, while interactions with macrophage TFs facilitate its H3K9 demethylase activities linked to its coactivator function (64,65,70,71). An intriguing yet puzzling result is that KDM1A depletion increased also the repressive H3K9me3 mark, in addition to H3K9me2. Indirect mechanisms may account for this, since H3K9me3 is not known to be a direct substrate for KDM1A. One possibility is that KDM1A cooperates with H3K9me demethylases including KDM4 family members, which have been reported to associate with both KDM1A and GPS2 in different contexts (31,39,65,72). Alternatively, changes of H3K9me3 upon depletion of KDM1A (or GPS2, increasing KDM1 recruitment to chromatin) may be due to altered activities of different histone H3K9 lysine methyltransferases (KMTs) (67) which operate in equilibrium with KDMs, consistent with related observations in other cellular contexts (66).

Our data also indicate that the KDM1A coactivator function and the identified GPS2-KDM1A antagonism is not abolished by inhibitors of the KDM1A demethylase activity directed towards H3K4me1/2. Whether this is limited to a subset of GPS2/IL4 target genes or a more general feature of KDM1A coactivation in macrophages remains to be clarified. The issue is of translational relevance as KDM1A is a promising epigenetic drug target for pharmacological inhibitors that are currently being developed for clinical applications (56). Thus, it is crucial to understand the mechanistic action and target range, but also the limitations of such inhibitors and their physiological consequences. KDM1A and its inhibitors have been extensively studied in different cellular contexts, revealing a variety of mechanisms of action at chromatin and enhancers, both dependent and independent of KDM1A's lysine demethylation activity (57–59,73). Although several mechanisms have recently also been identified in M1-type macrophages (60–63) (74), they seem to be distant from those discovered in our study. It is currently not clear whether this reflects fundamental mechanistic differences of KDM1A action in M1 versus M2 contexts or rather alternative mechanisms that operate in both contexts. However, any of the mechanisms should be affected by the different expression levels and functions of TFs and coregulators, including KDMs and KMTs, in either signaling context. M1 and M2 signaling pathways crosstalk with each other (16,75) and must be further addressed by integrating synergistic and antagonistic coregulator networks such as those identified here.

## DATA AVAILABILITY

RNA-seq, ChIP-seq, CUT&Tag, ATAC-seq and 4C-seq data generated in this study have been deposited at NCBI Gene Expression Omnibus (GEO) and the accession number is GSE184884. The relevant ChIP-seq and RNA-seq were from GSE130383, GSE110465, GSE66774 and GSE106706 series. Other data are available from the corresponding authors upon request.

## Supplementary Material

gkac1230_Supplemental_File
