# Supplementary material for: Antagonistic action of GPS2 and KDM1A at enhancers governs alternative macrophage activation by interleukin 4
Source: Nucleic Acids Res. 2023 Jan 5;51(3):1067–86. doi: 10.1093/nar/gkac1230 (PMC9943668; doi:10.1093/nar/gkac1230)
Supplement: gkac1230_Supplemental_File [file gkac1230_supplemental_file.pdf]

SUPPLEMENTARY INFORMATION

Antagonistic action of GPS2 and KDM1A at enhancers governs alternative macrophage activation by interleukin 4

Huang et al.

Supplementary Figures S1-S9

Supplementary Tables I-III

Supplementary Figure S1

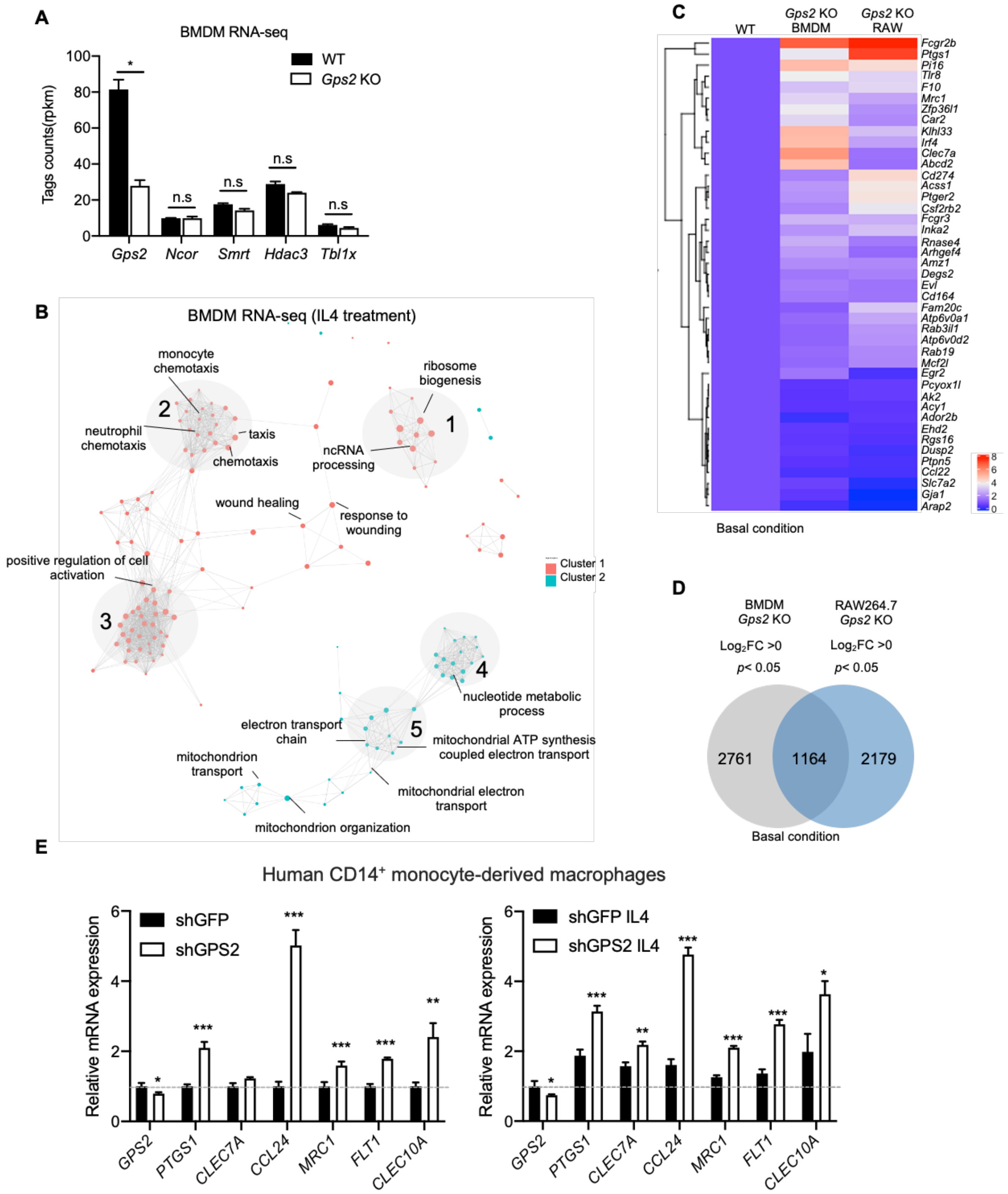

Supplementary Figure S1. Transcriptomic comparison of GPS2 depletion in BMDMs and RAW cells.

(**A**) RNA-seq tag counts (RPKM) of *Gps2, Ncor, Smrt, Hdac3 and Tbl1x* gene expression in WT vs. *Gps2* KO BMDMs. (**B**) Network of the top enriched GO terms from pathway analysis of significantly up-regulated (cluster 1) and down-regulated (cluster 2) genes in WT vs. *Gps2* KO BMDMs. (**C**) Heat map of top- regulated genes in *Gps2* KO RAW and BMDM cells; (**D**) Venn diagram showing the overlap of GPS2-regulated transcriptome signatures in BMDMs and RAW cells. (**E**) RT-qPCR analysis of mRNA expression under GPS2 knockdown with IL4 treatment in human $CD14^+$ monocyte-derived macrophages (n=3). *CLEC10A* is related to mouse *Mgl2*. Unpaired t test was used to determine data significance. All data were represented as mean ± s.e.m. *P < 0.05, **P < 0.01, ***P < 0.001.

Supplementary Figure S2

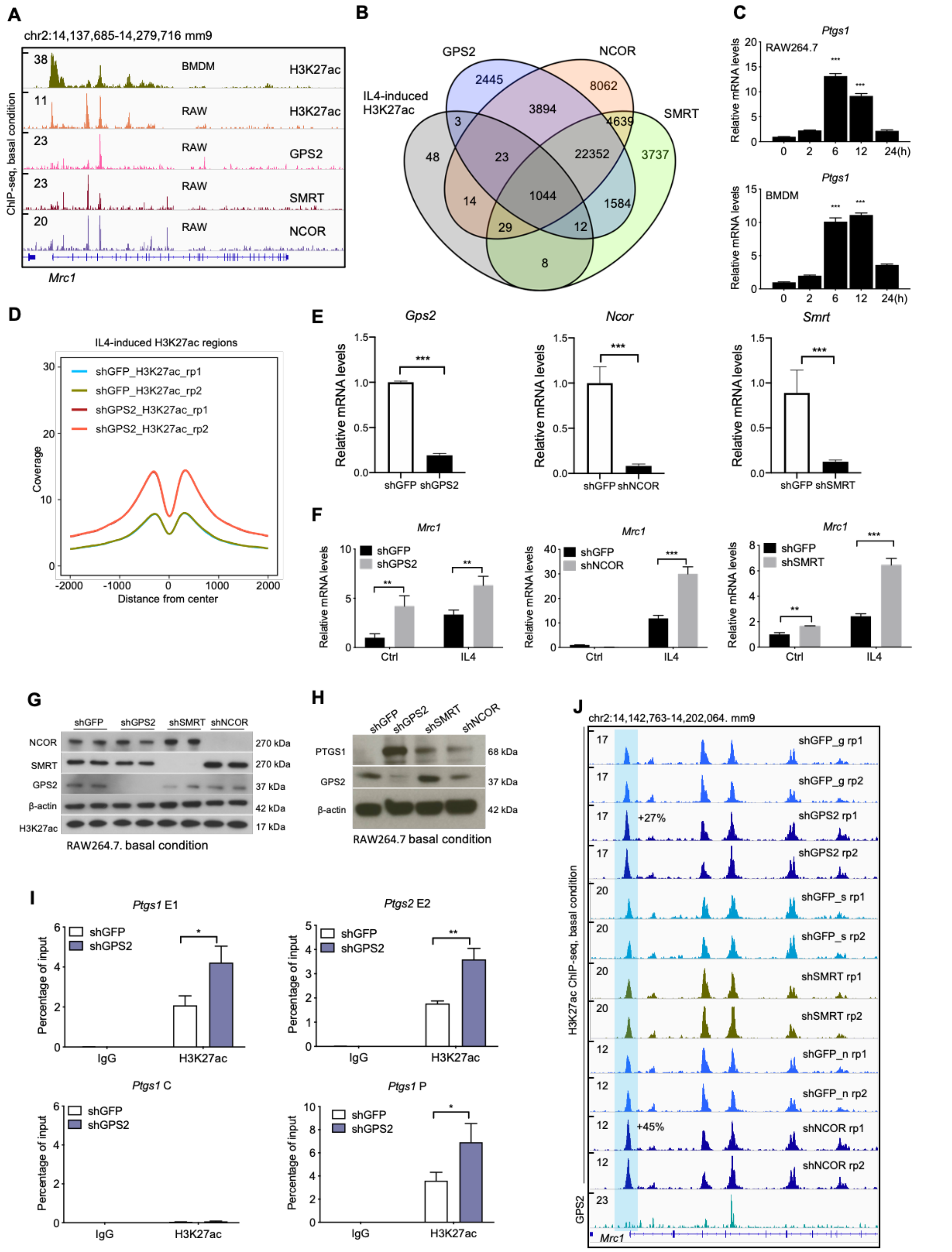

Supplementary Figure S2. GPS2 works together with NCOR and SMRT in controlling IL4-responsive gene expression.

(**A**) IGV genome browser tracks representing H3K27ac and recruitment of GPS2, SMRT and NCOR at the *Mrc1* locus. (**B**) Venn diagram showing the overlap of the corepressor complex subunit cistrome with the IL4-induced H3K27ac regions. (**C**) RT-qPCR analysis of the time-dependent expression of *Ptgs1* mRNA in RAW cells and BMDMs upon IL4 treatment (n=3). One-way ANOVA test was used for multiple comparisons. (**D**) H3K27ac peak coverage in the IL4-induced H3K27ac regions upon GPS2 depletion (n=2). (**E**) RT-qPCR showing the mRNA expression of *Gps2*, *Ncor,* and *Smrt* upon lentivirus-mediated shRNA knockdown in RAW cells (n=3). Unpaired t test was used to determine data significance; (**F**) RT-qPCR analysis of *Mrc1* expression in the GPS2, NCOR and SMRT knockdown cells under IL4 treatment (n=3); (**G**) Western blot analysis of GPS2, NCOR, and SMRT proteins in the indicated knockdown cells (n=2); (**H**) Western blot analysis of PTGS1 protein in the indicated knockdown cells; (**I**) ChIP-qPCR validation of H3K27ac changes in GPS2 knockdown cells (n=3). *Ptgs1* C, P, E1, E2 representing the PCR primers used for *Ptgs1* negative control region, promoter, and enhancers. (**J**) IGV genome browser tracks representing H3K27ac at the *Mrc1* locus upon the indicated shRNA knockdowns (n=2). Unpaired t test was used to determine data significance. All data were represented as mean ± s.e.m. $^{*}P < 0.05$, $^{**}P < 0.01$, $^{***}P < 0.001$.

Supplementary Figure S3

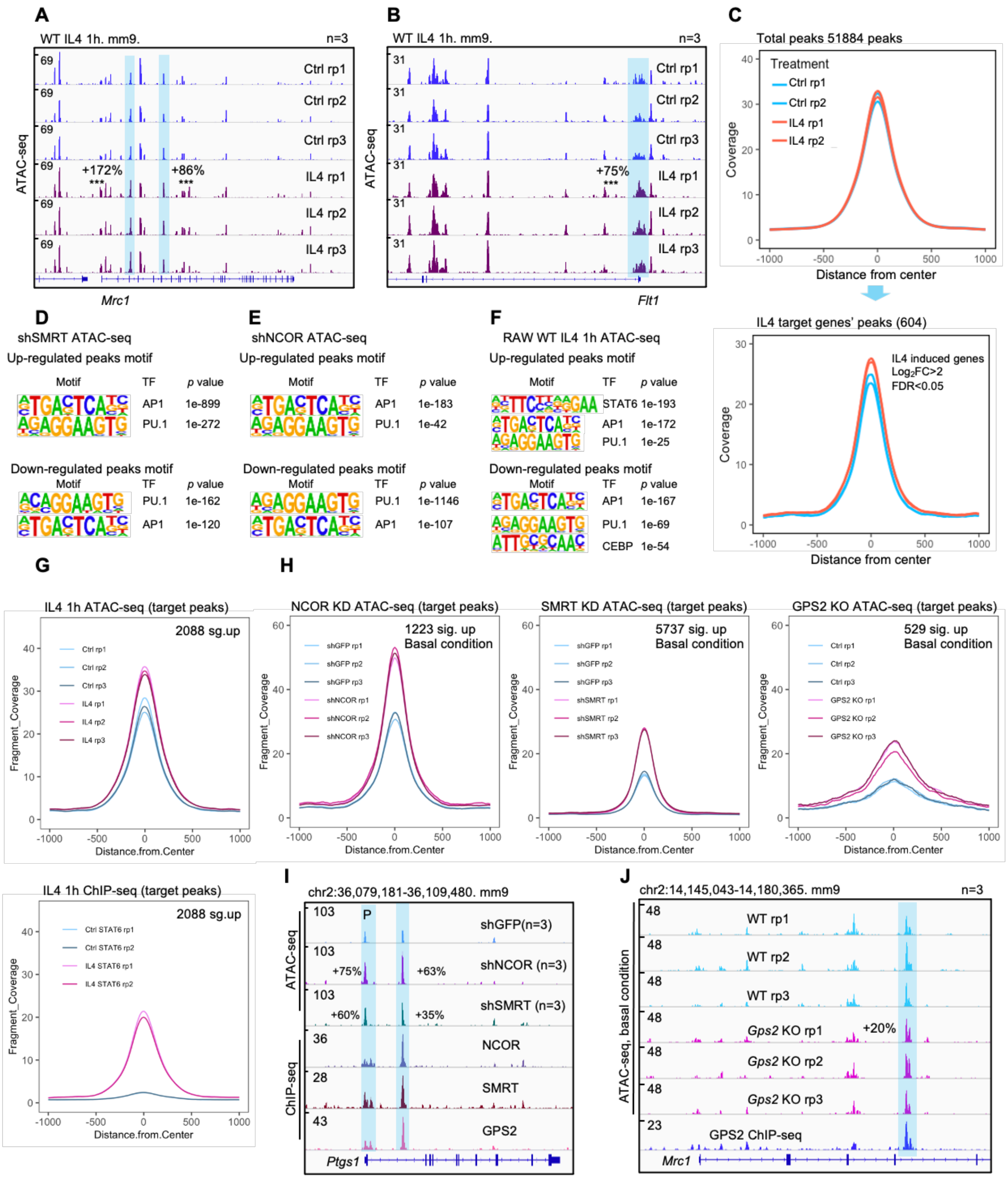

Supplementary Figure S3. GPS2, SMRT and NCOR restrict chromatin access at IL4 target gene loci.

(**A**-**B**) Genome browser tracks representing the ATAC-seq signal changes upon IL4 treatment at the (**A**) *Mrc1* and (**B**) *Flt1* loci in RAW cells. Regions with increased chromatin accessibility were highlighted and quantified (control vs. IL4). (**C**) Peak coverage representing ATAC-seq genome-widely (upper panel) and at IL4-inducible gene loci (lower panel) under both control and IL4 treatment in RAW cells; (**D**-**E**) TF motif analysis of up- and down-regulated peaks upon depletion of NCOR or SMRT in RAW cells; (**F**) TF motif analysis of up- and down-regulated peaks upon IL4 vs. control in RAW cells. (**G**) Peak coverage representing chromatin accessibility (ATAC-seq) changes in IL4-induced regions (upper panel) and the corresponding STAT6 enrichment (lower panel) in the same regions. (**H**) Peak coverage representing chromatin accessibility (ATAC-seq) changes (significantly upregulated regions) upon depletion of NCOR, SMRT or GPS2. (**I**) IGV genome browser tracks representing ATAC-seq peaks in the indicated knockdown cells and ChIP-seq peaks at the *Ptgs1* locus. (**J**) IGV genome browser tracks representing ATAC-seq peaks in WT and *Gps2* KO cells along with GPS2 ChIP-seq peaks at the *Mrc1* locus.

Supplementary Figure S4

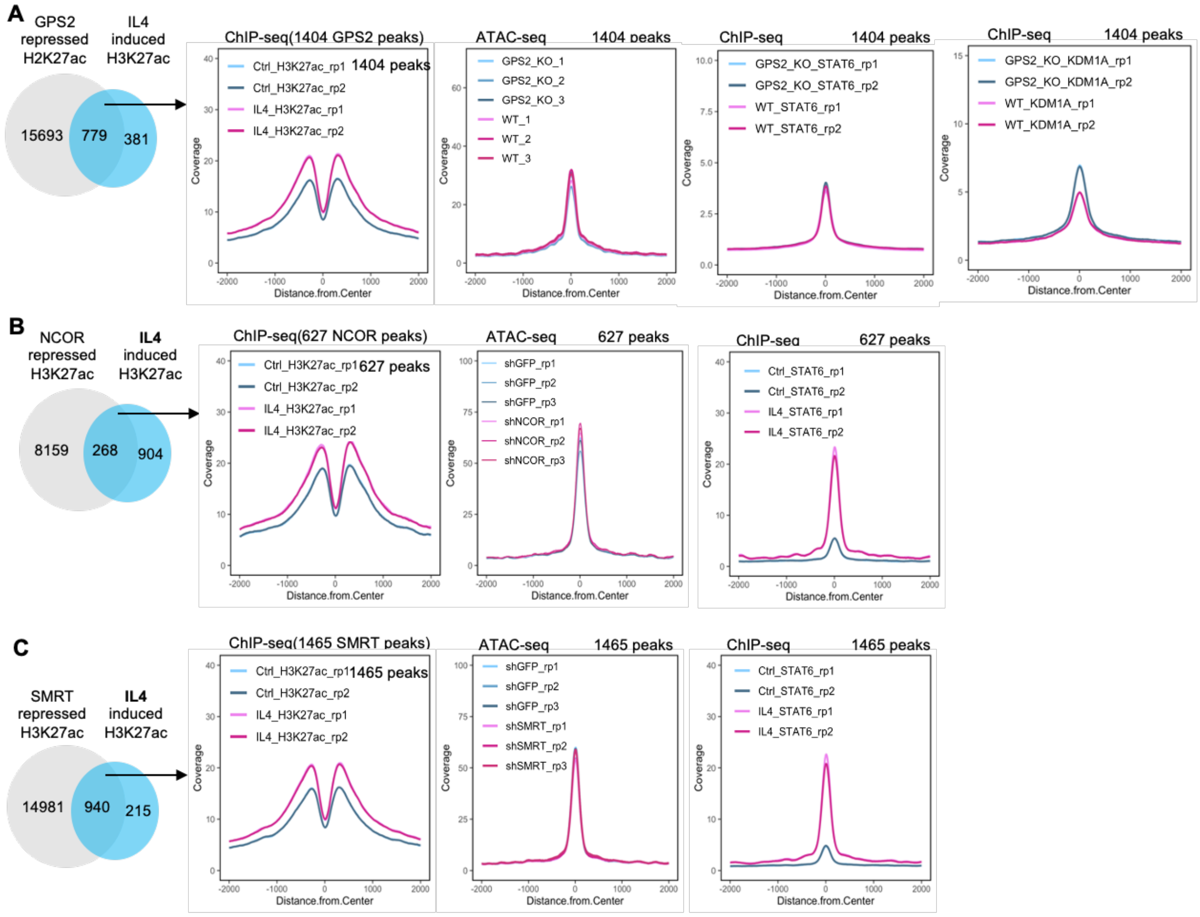

Motif	TF	*p* value
ATGACTCATC	AP1	1e-404
AGAGGAAGTG	PU.1	1e-132
ATTGCGCAAC	CEBP	1e-25
ACTTCCTAAGAA	STAT6	1e-22

Motif	TF	*p* value
ATGACTCATC	AP1	1e-137
AGAGGAAGTG	PU.1	1e-66
ACTTCCTAAGAA	STAT6	1e-11
ATTGCGCAAC	CEBP	1e-10

Motif	TF	*p* value
ATGACTCATC	AP1	1e-465
AGAGGAAGTG	PU.1	1e-142
ATTGCGCAAC	CEBP	1e-29
ACTTCCTAAGAA	STAT6	1e-19

Supplementary Figure S4. Comparison the DNA accessibility changes upon depletion of GPS2, NCOR or SMRT at IL4-inducible regions.

(**A**) Peak coverage representing the overlap of the IL4-inducible and GPS2-repressed H3K27ac regions. Venn diagram was used to select the common regions (left panel). GPS2 peaks were intersected from these regions. Peak coverages were derived from STAT6, KDM1A ChIP-seq and ATAC-seq in GPS2 KO cells (right panel). (**B-C**) Peak coverage representing the overlap of the IL4-inducible and NCOR/SMRT-repressed H3K27ac regions. Venn diagram was used to select the common regions (left panel). NCOR (**B**) and SMRT (**C**) peaks were intersected from these regions. Peak coverages were derived from STAT6 and H3K27ac ChIP-seq in these regions under IL4 treatment (**B-C**). ATAC-seq changes were plotted in these regions upon depletion of NCOR (**B**) or SMRT (**C**) (right panel). (**D**) TF motif analysis of GPS2, NCOR or SMRT-repressed IL4 target regions in RAW cells (related to **A-C**).

Supplementary Figure S5

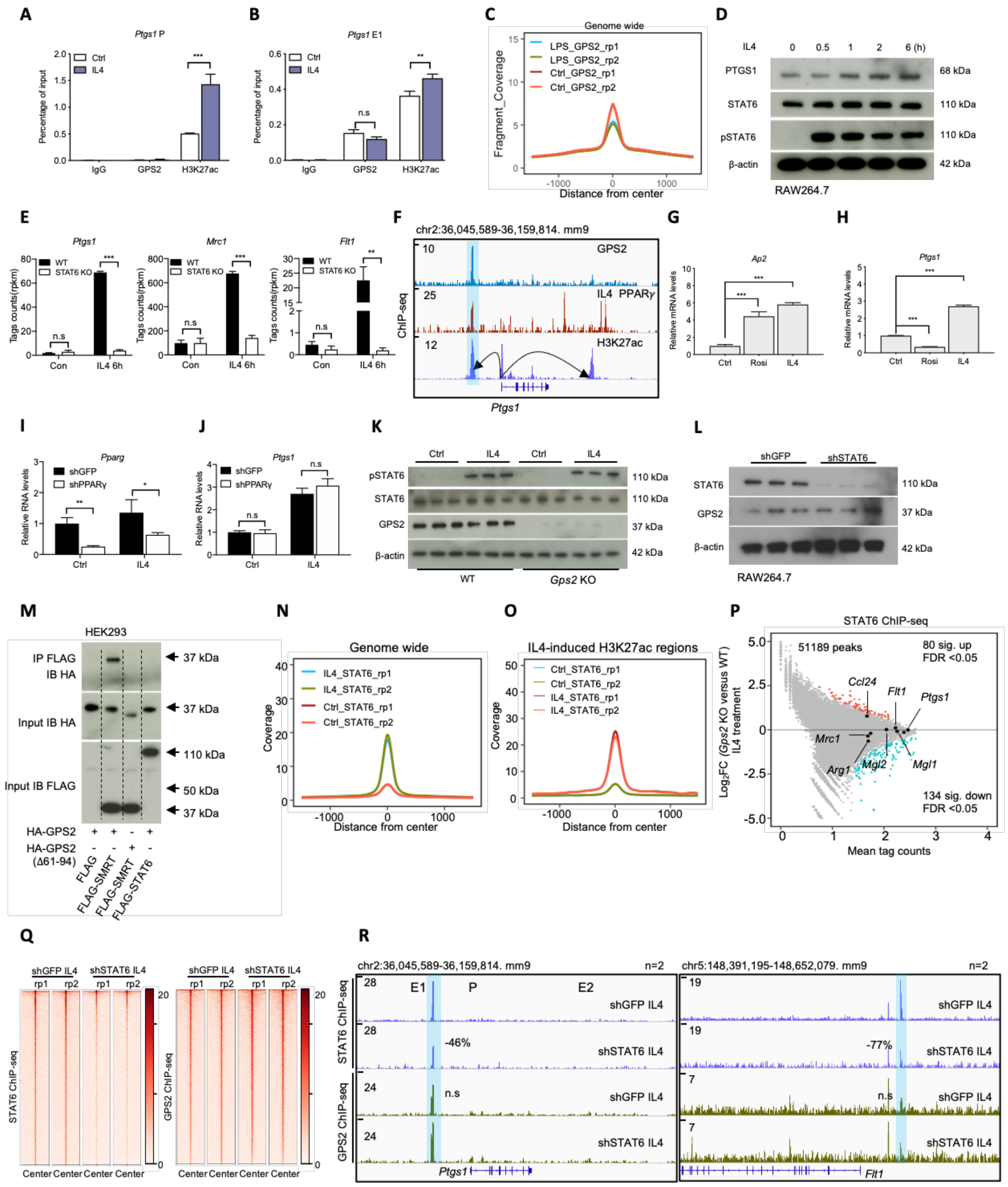

Supplementary Figure S5. Interplay of GPS2 with STAT6 in IL4-dependent gene regulation.

(**A**-**B**) ChIP-qPCR of GPS2 and H3K27ac at *Ptgs1* (**A**) promoter (P) and (**B**) enhancer (E1) in control vs. IL4-treated RAW cells (n=3). (**C**) Peak coverage representing the genome-wide GPS2 binding changes under LPS treatment in RAW cells (n=2). ChIP-seq data were from GSM4848601 to GSM4848604. (**D**) Western blot analysis of STAT6, pSTAT6, and PTGS1 protein levels with IL4 treatment (0, 30 min, 1 h, 2 h, 6 h); (**E**) Tag counts (RPKM) of *Ptgs1*, *Mrc1*, and *Flt1* from RNA-seq in WT vs. *Stat6* KO BMDMs upon IL4 treatment. RNA-seq data were from GSE106706. (**F**) IGV genome browser tracks representing PPARγ and GPS2 occupancy in control and IL4-treated BMDMs. PPARγ ChIP-seq data were from GSM2867738 and GSM2867739. GPS2 ChIP-seq data were from GSM1631866. (**G**-**H**) RT-qPCR analysis of PPAR*γ* target gene expression (**G**) *Ap2* and (**H**) *Ptgs1* in control cells and upon treatment with Rosiglitazone or IL4 (n=3). (**I**-**J**) RT-qPCR analysis of (**I**) *Pparg* and (**J**) *Ptgs1* expression in control vs. IL4-treated WT and shPPARγ RAW cells (n=3). (**K**) Western blot analysis of STAT6, pSTAT6, and GPS2 expression in WT and *Gps2* KO RAW cells in control vs. IL4 condition (n=3). (**L**) Western blot analysis of GPS2 and STAT6 protein levels in shSTAT6 RAW cells (n=3). (**M**) Co-immunoprecipitation of HA-GPS2 with FLAG-tagged STAT6 in HEK293 cells. FLAG-SMRT (aa 1–300, GPS2-binding domain). HA-GPS2 (Δ aa 61–94, deletion of the SMRT-binding domain) served as a negative control. (**N**-**O**) Peak coverage representing the (**N**) genome-wide and (O) IL4-induced binding changes of STAT6 in IL4-treated vs. control RAW cells (n=2). (**P**) MA plot showing STAT6 peak changes in *Gps2* KO vs. WT cells. Significant up-regulated and down-regulated peaks were highlighted in red and blue. The key IL4 targets gene peaks were labelled. (**Q**) Genome-wide heatmap of STAT6 and GPS2 ChIP-seq in shGFP control and shSTAT6 RAW cells under IL4 treatment (n=2). (**R**) IGV genome browser tracks representing the GPS2 and STAT6 recruitment at the *Ptgs1* and *Flt1* loci in shSTAT6 RAW cells or *Gps2* KO cells under IL4 treatment (n=2). Unpaired t test was used to determine data significance. All data were represented as mean ± s.e.m. *P < 0.05, **P < 0.01, ***P < 0.001.

Supplementary Figure S6

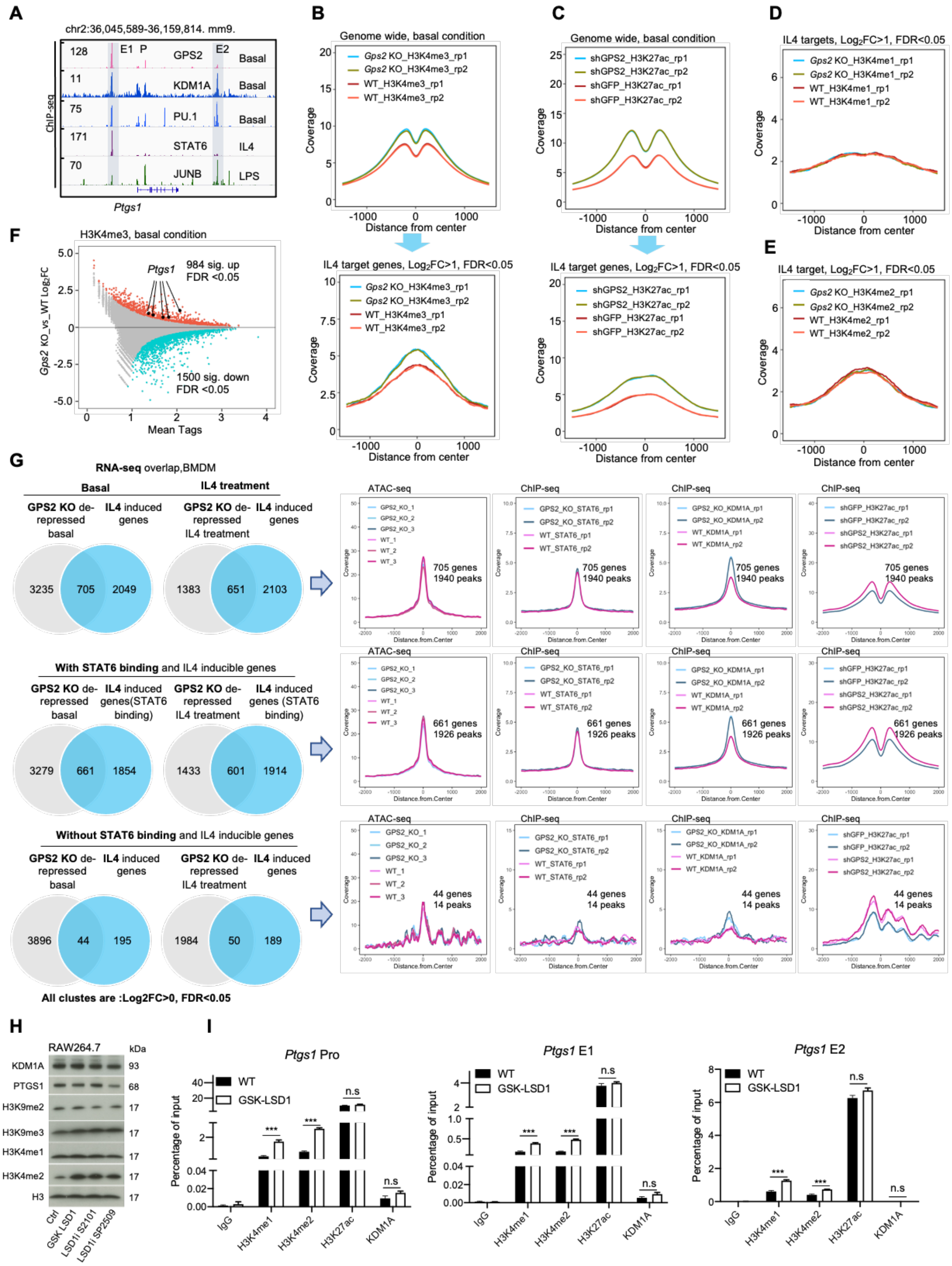

Supplementary Figure S6. Epigenetic changes at IL4 target gene loci upon depletion of GPS2 or upon treatment with KDM1A inhibitors.

(**A**) IGV genome browser tracks showing GPS2, KDM1A, PU.1, STAT6 and JUNB recruitment at the *Ptgs1* locus. PU.1 and JUNB ChIP-seq data were from GSM4848530 and GSM4848586. (**B**-**C**) Peak coverage of (**B**) H3K4me3 and (**C**) H3K27ac in control (WT or shGFP) and GPS2-depleted (CRISPR KO or shRNA knockdown) RAW cells both genome-widely (upper panels) and at IL4 target gene loci (lower panels). (**D**-**E**) Peak coverage of (**D**) H3K4me1 and (**E**) H3K4me2 at IL4 target gene loci in GPS2 KO cells. (**F**) MA plot showing the H3K4me3 log2 fold changes in GPS2 KO vs. WT RAW cells. Significantly up- and down-regulated peaks were highlighted in red or blue. Up-regulated peaks within the *Ptgs1* gene locus were labelled. (**G**) Comparison the GPS2- repressed and IL4-induced genes with or without STAT6 binding sites in basal and IL4 condition. Venn diagram was done from the GPS2 KO RNA-seq (left panels). Peak coverages were derived from the individual STAT6, KDM1A, H3K27ac ChIP-seq and ATAC-seq for the selected regions (right panels). (**H**) Western blot analysis of PTGS1, KDM1A and indicated histone modifications upon treatment with KDM1A inhibitors. (**I**) ChIP-qPCR of KDM1A, H3K27ac and H3K4me1/2 enrichment at the *Ptgs1* promoter (P) and enhancers (E1, E2), in RAW cells treated with the KDM1A inhibitor GSK-LSD1 (n=3).

Supplementary Figure S7

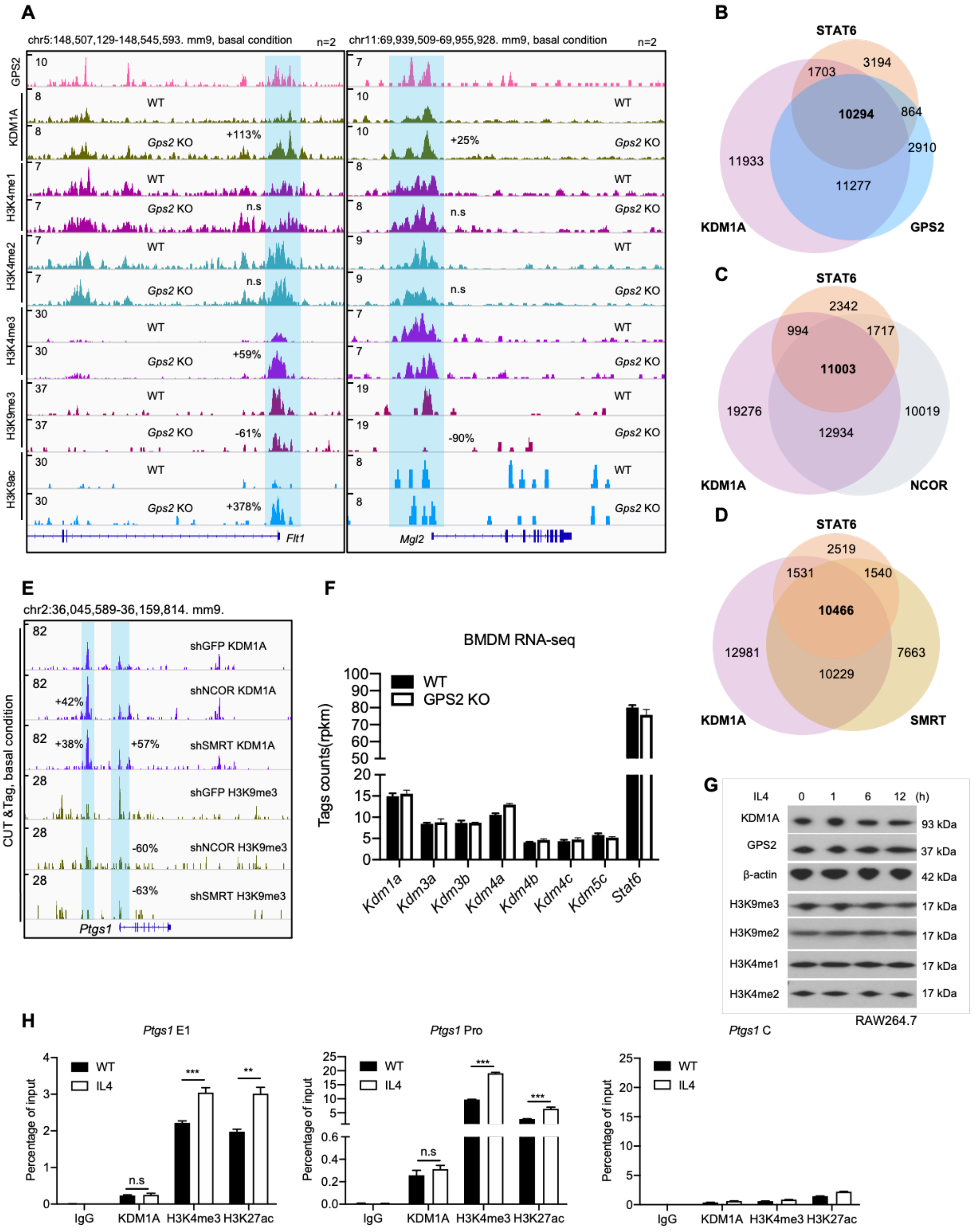

Supplementary Figure S7. Epigenetic links between GPS2 and KDM1A.

(**A**) IGV genome browser tracks showing KDM1A recruitment along with the indicated histone marks in WT and *Gps2* KO cells at the *Flt1* and *Mgl2* loci. (**B-D**) Venn diagram analysis of cistromes for KDM1A, STAT6 and the corepressor complex subunits GPS2 (**B**), NCOR (**C**), and SMRT (**D**) in RAW cells. (**E**) IGV genome browser tracks showing effects of NCOR or SMRT depletion on KDM1A recruitment and H3K9me3 levels at the *Ptgs1* locus. (**F**) RNA-seq tag counts (RPKM) determining relative gene expression of the indicated lysine demethylases in WT vs. *Gps2* KO BMDMs. (**G**) Western blot analysis of GPS2, KDM1A and the indicated histone modifications upon IL4 treatment (0, 1 h, 6 h, 12 h). (**H**) ChIP-qPCR of KDM1A, H3K27ac and H3K4me3 enrichment at the *Ptgs1* enhancer (E1), promoter (P), and control region (C), in control vs. IL4-treated (1 h) RAW cells (n=3).

Supplementary Figure S8

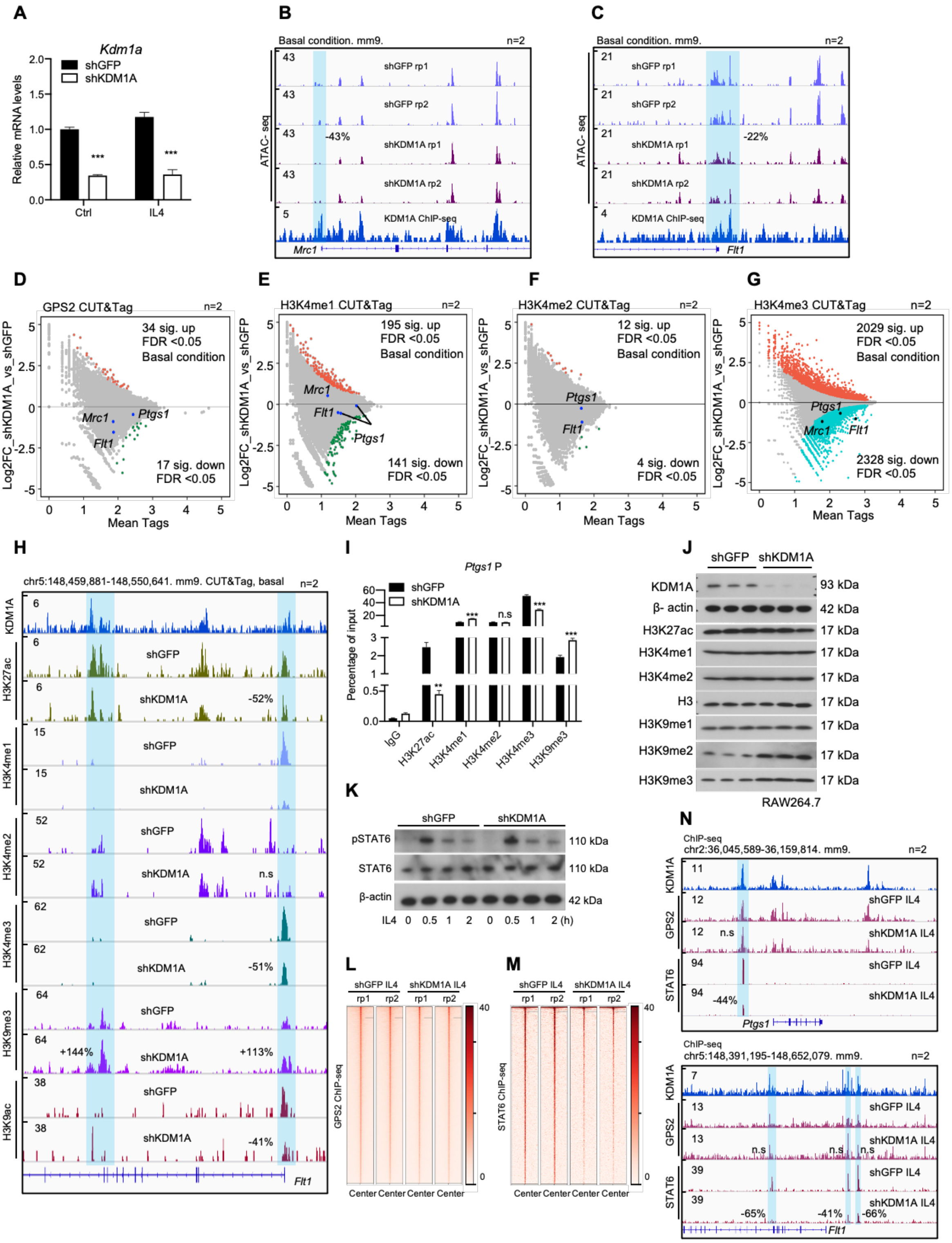

Supplementary Figure S8. Epigenetic changes at IL4 target gene loci upon KDM1A depletion.

(**A**) RT-qPCR analysis of *Kdm1a* expression KDM1A-depleted in control vs. IL4-treated RAW cells (n=3). (**B**-**C**) IGV genome browser tracks of ATAC-seq in shGFP and shKDM1A RAW cells along with KDM1A ChIP-seq peaks at the *Mrc1* (**B**) and *Flt1* (**C**) loci. (**D**-**G**) MA plots representing GPS2, H3K4me1, H3K4me2 and H3K4me3 peaks in shKDM1A vs. shGFP RAW cells. Significantly up- and down-regulated peaks were highlighted in red and green in each plot. Peaks within featured gene loci were labelled. (**H**) IGV genome browser tracks of KDM1A recruitment along with the indicated histone modifications at the *Flt1* locus in shGFP (control) and shKDM1A knockdown RAW cells. (**I**) ChIP-qPCR analysis of the indicated histone modifications at the *Ptgs1* promoter (P) (n=3). (**J**) Western blot analysis of total KDM1A protein levels along with the indicated histone modifications in shGFP vs. shKDM1A RAW cells (n=3). (**K**) Western blot analysis of total STAT6 and p-STAT6 protein levels in shGFP vs. shKDM1A RAW cells (n=3). (**L**) Genome-wide heatmap of GPS2 ChIP-seq in shGFP vs. shKDM1A RAW cells under IL4 treatment (n=2); (**M**) Genome-wide heatmap of STAT6 ChIP-seq in shGFP vs. shKDM1A RAW cells under IL4 treatment (n=2). (**N**) IGV genome browser tracks of GPS2 and STAT6 ChIP-seq in shGFP and shKDM1A RAW cells under IL4 treatment at the *Ptgs1* and *Flt1* loci. Unpaired t test was used to determine data significance. All data were represented as mean ± s.e.m. *P < 0.05, **P < 0.01, ***P < 0.001.

Supplementary Figure S9

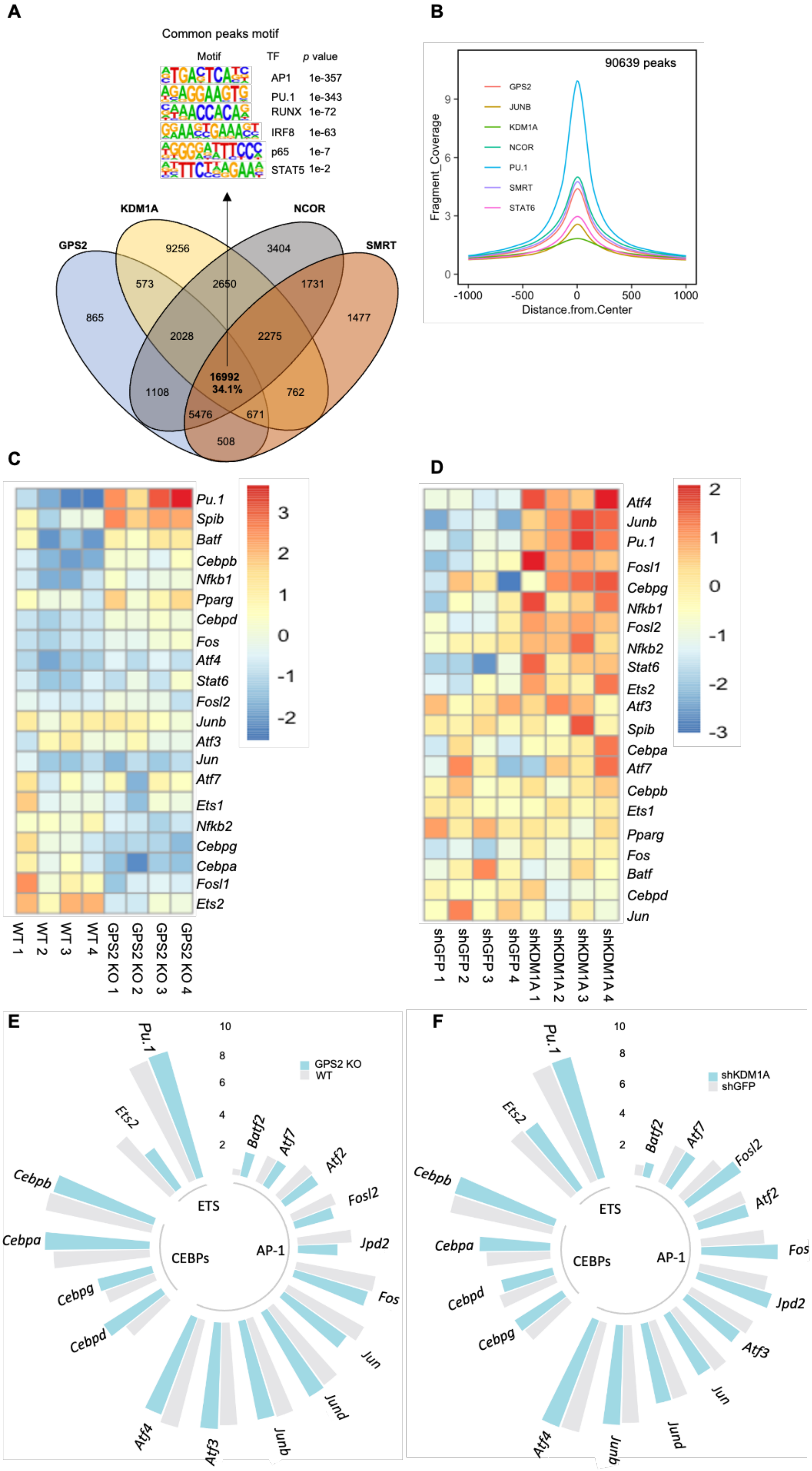

Supplementary Figure S9. Analysis of candidate target TFs for GPS2 and KDM1A.

(**A**) Venn diagram showing the overlap of GPS2, NCOR, SMRT, and KDM1A cistromes in RAW cells. TF motif analysis for indicated majority of common peaks is highlighted. (**B**) Peak coverage representing the genome-wide co-occupancy of the corepressor complex subunits, AP1(JUNB), PU.1, STAT6, and KDM1A. JUNB, and PU.1 ChIP-seq were from GSM4848586 and GSM4848528. (**C-D**) Transcriptome-based TF activity analysis: Heat maps representing the change in the activity of TFs based on the expression of their target genes upon depletion of either GPS2 in BMDMs (**C**) or KDM1A in RAW cells. (**E-F**) Circular plot representing the relative mRNA expression of selected TF family members upon depletion of GPS2 in BMDMs (**E**) or KDM1A in RAW cells (**F**).

Supplementary Table I

PCR primers used in this study

RT-qPCR primers	Forward Primer (5’-3’)	Reverse Primer (5’-3’)
Gps2	ACCAGCTTCTCGGACTCATCTTCT	GAGGGTGGGCTGGAGCTCTCT
Ncor	TGGATCCTGCTGCTGCTTACCT	GGCTGCTCTCGTGGGGACAGT
Smrt	GCCCTTAGTCCTAGGTGTGG	TTGTACAGAGGCGTGTGGGA
Ptgs1	CTTAGGCCACGGGGTAGA	TTTCCCATCCTTGAAGAGCCG
Ptgs2	GTTCATCCCTGACCCCCAAG	TTTAAGTCCACTCCATGGCCC
Clec7a	AACCACAAGCCCACAGAATCA	AGGTAACAGCTCTTCCCATGC
Ccl24	GGCAGGGGTCATCTTCATCAC	CTTGGCCCCTTTAGAAGGCT
Mrc1	GAAGATCCACTCTGGGCCAT	AGATTAAAAATTGCCTCGCGTCCAA
Mgl1	TTTGAGAAAGGCTTTAAGAACTGGG	GACCGGGTAGGAGGATTCCA
Mgl2	AGTCTCCAAAGTTTGCTCTAATTCC	AGGAGGTGGGTCCAAGAGAG
Arg1	CATTTGGGTGGATGCTCACA	TGGTACATCTGGGAACTTTCCTTT
Stat6	CAATGGTCCTGGTCCAAGTGA	CCAGGCTTTCACACCTCTCCTG
Ap2	TGAAAGAAGTGGGAGTGGGC	CCAGCTTGTCACCATCTCGT
Pparg	CGCGGAAGAAGAGACCTGG	ACCGCTTCTTTCAAATCTTGTCTG
Kdm1a	CTTCTGGAAGCCAGGGATCG	GAACAGCTTGTCCATTGGCT
Flt1	TACCTCACCGTGCAAGGAAC	AAGGAGCCAAAAGAGGGTCG
GPS2	GGCGAAAGGAACAGAGTGAC	AGCACTTGGGGTCCAAACAT
PTGS1	CAATGAGTACCGCAAGAGGTTTG	GTAGAACTCCAACGCATCAATGTC
KDM1A	TACTGTCGTGCCTGGGTCTG	GTACTCTACCTTCGCCCGC
CELC7A	GGAAGCAACACATTGGAGAATGG	AGAACCCCTGTGGTTTTGACA
CCL24	ACATCATCCCTACGGGCTCT	CTTGGGGTCGCCACAGAAC
MRC1	GGGAAAGGTTACCCTGGTGG	GTCAAGGAAGGGTCGGATCG
FLT1	TCATTCCGAAGCAAGGTGTGA	AGAGTCAGCCACAACCAAGG
CLEC10A	GAACGGCAGGCAGGGGTATC	AGGCATTGTTGTTGAGAGTAGCC
RPS14	GGCAGACCGAGATGAATCCTCA	CAGGTCCAGGGGTCTTGGTCC
GAPDH	GTCTCCTCTGACTTCAACAGCG	ACCACCCTGTTGCTGTAGCCAA
**ChIP-qPCR primers**	**Forward Primer (5’-3’)**	**Reverse Primer (5’-3’)**
ChIP *Ptgs1* E1	TTCTCCTCTGCCCACAACAC	GCATTCTGGAGGTGGCTTCT
ChIP *Ptgs1* E2	AACCAATGTGGCTCATGGAGA	GACGCTTCTTGTCGATGCTC
ChIP *Ptgs1* Pro	AAAATTCCAAGTCCTGCGCC	AGGTAGGCCCTAGCCATTCT
ChIP *Ptgs1* C	GACACATTGGCAGGACCAGA	CCAGGCCCCAGGATCAAAAT
**4C-seq primers:**	**Forward Primer (5’-3’)**	**Reverse Primer (5’-3’)**
Ptgs1 pro	CTCAGACCGTAACTCAC**GATC**	GCGGAGCCTTGAACG**CTAG**

Supplementary Table II

Antibodies used in this study

Antibody	Manufacturer	Item number	Application
FLAG	Sigma-Aldrich	F7425	WB
GPS2	Homemade	N/A	WB/ChIP-seq/CUT&Tag
H3	Abcam	ab1791	WB
H3K27ac	Abcam	ab4729	WB/ChIP-seq/CUT&Tag
H3K4me1	Abcam	ab176877	WB/ChIP-seq/CUT&Tag
H3K4me2	Abcam	ab32356	WB/ChIP-seq/CUT&Tag
H3K4me3	Abcam	ab8580	WB/ChIP-seq/CUT&Tag
H3K9ac	Sigma-Aldrich	06-942	ChIP-seq/CUT&Tag
H3K9me2	Abcam	ab1220	WB
H3K9me3	Abcam	ab8898	WB/ChIP-seq/CUT&Tag
HA	BioLegend	901513	WB
KDM1A	Abcam	ab17721	WB/ChIP-seq/CUT&Tag
NCOR	Bethyl	A301-145A	WB/ChIP-seq
Pol II	Biolegend	8WG16	ChIP-seq
PTGS1	Sigma-Aldrich	AV41836	WB
p-STAT6	Thermo Fisher	700247	WB/ChIP-seq
SMRT	Bethyl	A301-147A	WB/ChIP-seq
STAT6	Cell Signaling	9362	ChIP-seq
STAT6	Sigma-Aldrich	S-6433	WB
β-actin	Abcam	ab8226	WB

Supplementary Table III

Public datasets used in this study

GSM number	Antibody	Type	Tissue	Treatment	GEO series
GSM4848501	H3K27ac	ChIP-seq	RAW264.7	shGFP_for_Gps2 rp1	GSE130383
GSM4848502	H3K27ac	ChIP-seq	RAW264.7	shGFP_for_Gps2 rp2	GSE130383
GSM4848503	H3K27ac	ChIP-seq	RAW264.7	shGPS2 rp1	GSE130383
GSM4848504	H3K27ac	ChIP-seq	RAW264.7	shGPS2 rp2	GSE130383
GSM4848505	H3K27ac	ChIP-seq	RAW264.7	shGFP_for_Smrt_H3K27ac_rp1	GSE130383
GSM4848506	H3K27ac	ChIP-seq	RAW264.7	shGFP_for_Smrt_H3K27ac_rp2	GSE130383
GSM4848507	H3K27ac	ChIP-seq	RAW264.7	shSMRT rp1	GSE130383
GSM4848508	H3K27ac	ChIP-seq	RAW264.7	shSMRT rp2	GSE130383
GSM4848530	PU.1	ChIP-seq	RAW264.7	LPS	GSE130383
GSM4848586	JUNB	ChIP-seq	RAW264.7	LPS	GSE130383
GSM4848601	GPS2	ChIP-seq	RAW264.7	Basal rp1	GSE130383
GSM4848602	GPS2	ChIP-seq	RAW264.7	Basal rp2	GSE130383
GSM4848603	GPS2	ChIP-seq	RAW264.7	LPS rp1	GSE130383
GSM4848604	GPS2	ChIP-seq	RAW264.7	LPS rp2	GSE130383
GSM2845662	STAT6	ChIP-seq	BMDM	Ctrl rp1	GSE106706
GSM2845663	STAT6	ChIP-seq	BMDM	Ctrl rp2	GSE106706
GSM2845664	STAT6	ChIP-seq	BMDM	IL4 rp1	GSE106706
GSM2845665	STAT6	ChIP-seq	BMDM	IL4 rp2	GSE106706
GSM2867738	PPARγ	ChIP-seq	BMDM	Basal	GSE110465
GSM2867739	PPARγ	ChIP-seq	BMDM	IL4	GSE110465
GSM1631862	H3K27ac	ChIP-seq	BMDM	Basal	GSE66774
GSM1631866	GPS2	ChIP-seq	BMDM	Basal	GSE66774
GSM2845727	N/A	RNA-seq	BMDM	*Stat6* KO Ctrl rp1	GSE106706
GSM2845728	N/A	RNA-seq	BMDM	*Stat6* KO Ctrl rp2	GSE106706
GSM2845725	N/A	RNA-seq	BMDM	*Stat6* KO IL4 6h 1	GSE106706
GSM2845726	N/A	RNA-seq	BMDM	*Stat6* KO IL4 6h 2	GSE106706
GSM2845707	N/A	RNA-seq	BMDM	BMDM Ctrl 1	GSE106706
GSM2845708	N/A	RNA-seq	BMDM	BMDM Ctrl 2	GSE106706
GSM2845709	N/A	RNA-seq	BMDM	BMDM Ctrl 3	GSE106706
GSM2845710	N/A	RNA-seq	BMDM	BMDM Ctrl 4	GSE106706
GSM2845703	N/A	RNA-seq	BMDM	BMDM IL4 6h 1	GSE106706
GSM2845704	N/A	RNA-seq	BMDM	BMDM IL4 6h 2	GSE106706
GSM2845705	N/A	RNA-seq	BMDM	BMDM IL4 6h 3	GSE106706
GSM2845706	N/A	RNA-seq	BMDM	BMDM IL4 6h 4	GSE106706
